# Individual Retinal Progenitor Cells Display Extensive Heterogeneity of Gene Expression

**DOI:** 10.1371/journal.pone.0001588

**Published:** 2008-02-13

**Authors:** Jeffrey M. Trimarchi, Michael B. Stadler, Constance L. Cepko

**Affiliations:** 1 Department of Genetics, Harvard Medical School, Boston, Massachusetts, United States of America; 2 Friedrich Miescher Institute for Biomedical Research, Basel, Switzerland; 3 Howard Hughes Medical Institute, Harvard Medical School, Boston, Massachusetts, United States of America; Katholieke Universiteit Leuven, Belgium

## Abstract

The development of complex tissues requires that mitotic progenitor cells integrate information from the environment. The highly varied outcomes of such integration processes undoubtedly depend at least in part upon variations among the gene expression programs of individual progenitor cells. To date, there has not been a comprehensive examination of these differences among progenitor cells of a particular tissue. Here, we used comprehensive gene expression profiling to define these differences among individual progenitor cells of the vertebrate retina. Retinal progenitor cells (RPCs) have been shown by lineage analysis to be multipotent throughout development and to produce distinct types of daughter cells in a temporal, conserved order. A total of 42 single RPCs were profiled on Affymetrix arrays. *In situ* hybridizations performed on both retinal sections and dissociated retinal cells were used to validate the results of the microarrays. An extensive amount of heterogeneity in gene expression among RPCs, even among cells isolated from the same developmental time point, was observed. While many classes of genes displayed heterogeneity of gene expression, the expression of transcription factors constituted a significant amount of the observed heterogeneity. In contrast to previous findings, individual RPCs were found to express multiple bHLH transcription factors, suggesting alternative models to those previously developed concerning how these factors may be coordinated. Additionally, the expression of cell cycle related transcripts showed differences among those associated with G2 and M, versus G1 and S phase, suggesting different levels of regulation for these genes. These data provide insights into the types of processes and genes that are fundamental to cell fate choices, proliferation decisions, and, for cells of the central nervous system, the underpinnings of the formation of complex circuitry.

## Introduction

One key question in developmental biology is how progenitor cells, cells that are still dividing and have not as yet chosen any particular cellular fate, are specified to generate a precise set of cell types. In the nervous system, this question is of further interest, since the formation of the proper neuronal circuitry often depends upon the generation of particular types of neurons in the appropriate location and with the correct timing. The exact mechanisms that control these processes in neural progenitor cells are not well understood at present, but are believed to involve some combination of extrinsic signaling pathways and intrinsic factors [1], [2], [3]. It has been noted that a fairly small number of signaling pathways are used iteratively in development, with very different outcomes, not only across tissues, but even within a single developing tissue [4], [5]. The distinct outcomes must be in large part due to differences among individual progenitor cells. However, to date, there has not been a comprehensive analysis of the differences among progenitor cells within any developing tissue. There have been a few studies that have begun to examine gene expression at a single cell level, but they were constrained either due to the small number of genes sampled [6], [7], [8] or due to the small number of cells profiled [9], [10]. Without an approach that involves a full complement of transcripts and many more cells, one cannot gain an appreciation of the contribution of progenitor cell heterogeneity to the production of the many types of progeny cells within a tissue. Beyond the obvious interest in this question for developmental biologists, therapeutic strategies reliant upon stem cells will need such information to direct stem cells into particular progenitor cell states.

The vertebrate retina has served as a model system for the development of the central nervous system (CNS). Although it contains only six major neuronal cell types and one glial cell type [11], further distinctions among the neurons relevant to circuitry and information transformations show that there is at least 50 types of cells in the mature retina [12]. Lineage analyses in several species have shown that these cell types are produced from a pool of multipotent progenitor cells throughout development [13], [14], [15], with even terminal divisions capable of giving rise to two very different cell types, such as a photoreceptor cell and an interneuron. [^3^H]-thymidine based birthdating studies have demonstrated that these retinal cell types are generated in overlapping intervals and with a conserved birth order [16], [17], [18]. The major output neuron, the retinal ganglion cell, is the first to be generated, followed by the onset of cone photoreceptor and horizontal cell (an interneuron) genesis shortly thereafter (reviewed in [19]). The appearance of another type of interneuron, the amacrine cell, occurs slightly later still, with rod photoreceptor cells, bipolar cells (another interneuron type) and Muller glia being the latest born retinal cell types (reviewed in [19].

The classical studies cited above set the stage for further analyses of RPCs. Among the key questions addressed through several experimental protocols was whether the RPCs were equivalent throughout development. To examine this possibility, mixing experiments where RPCs from different development stages were either co-cultured with cells of different ages [20], [21] or transplanted [22], demonstrated that RPCs were not equivalent throughout development. While environmental factors could influence the relative proportions of the different cell types produced, these signals could not induce the RPCs to generate temporally inappropriate cell types [20], [21], [22]. In addition, culture of isolated RPCs led to the formation of clones with the composition of those formed *in vivo* [23]. These findings led to the idea that RPCs pass through a series of competence states where these cells can only produce a subset of retinal cell types [1]. Additionally, these states are intrinsically defined. An attractive hypothesis for how this is achieved involves the dynamic expression of different combinations of transcription factors at distinct times [1]. The competence model for retinal development received strong support from studies in the ventral nerve cord of *Drosophila melanogaster* [24]. In this organism, the temporal order of neuronal progeny produced by neuroblasts is driven by the well defined sequential expression of the transcription factors Hunchback (Hb), Krüppel (Kr), Pdm and Castor [24]. Experiments in which the expression of Hb was maintained beyond its normal window resulted in an extension of the early competence state and a corresponding increase in the number of early born neurons generated [25]. When this enforced expression of Hb was removed, neuroblasts expressed Kr and continued on to the later competence states [25].

The genes that define the particular RPC competence states as well as those that regulate the transitions between them are only just beginning to be identified. Large scale gene expression profiling studies have been utilized as a first step toward revealing all of the potential transcripts involved in RPC biology [26], [27], [28], [29]. However, previous microarray and SAGE based screening studies focused on the entire retina, thus homogenizing the tissue and potentially obscuring underlying heterogeneity [27], [28], [29]. Nonetheless, a handful of genes have been identified as being expressed in subsets of RPCs [30], [31], [32], [33], [34], [35] including some genes that displayed a temporally restricted expression pattern [27]. However, it was unclear from these studies how similar or different individual RPCs were relative to each other, both across developmental time and at specific timepoints [27].

To begin to assess the degree of gene expression heterogeneity among RPCs, individual retinal cells were harvested from six different developmental timepoints ranging from embryonic day 12.5 (E12.5) through postnatal day 0 (P0). Included within this population were newborn neurons, as well as a few neurons further along in their differentiation program. The transcriptomes from these cells were used to classify each cell as an RPC, a cell in transition between an RPC and a neuron, or a neuron. In total, 64 cells were profiled on Affymetrix mouse 430 2.0 oligonucleotide arrays, encompassing 36 RPCs, 6 transitional cells and 22 neurons. Examination of the gene expression profiles from these cells revealed an extensive amount of heterogeneity among RPCs, even among those RPCs isolated from the same day of development. In particular, transcription factors were responsible for a significant amount of the observed heterogeneity. Cell cycle regulators also accounted for some of the differences in gene expression among RPCs. Interestingly, genes that have been more associated with the G2 phase of the cell cycle displayed more heterogeneity than those that have been linked with the G1 phase, pointing to possible differences in how these genes are regulated. A more in depth examination of the G2 RPCs revealed additional genes that may be correlated with the production of postmitotic neurons from RPCs. Surprisingly, it appears that different RPCs might be using different genes to regulate exit from the cell cycle. *In situ* hybridizations on retinal cryosections and dissociated retinal cells allowed for validation and quantitative extension of the observed heterogeneity in the microarray data. The results from this study provide the most comprehensive and in depth examination of dynamic gene expression patterns of individual cells in a developing tissue, and suggest that progenitor cell heterogeneity plays a major role in the production of the distinct cell types comprising complex tissues.

## Materials and Methods

### Single cell collection and PCR based cDNA amplification

Single retinal cells were isolated, their mRNAs were reverse transcribed, and the resulting cDNAs were PCR amplified exactly as described previously [36]. Briefly, retinas were dissected from CD-1 (Charles River Laboratories) mouse embryos and dissociated to single cells through the combination of papain (Worthington Biochemical) digestion and gentle trituration. Cells were washed, pelleted and resuspended in PBS (pH 7.4) containing 0.1% BSA. Individual retinal cells were harvested using a mouth pipette and a capillary (Sigma) drawn into a fine glass needle. Single cells were captured by allowing capillary action to draw them into the needle. To ensure that only one cell was picked, each collected cell was expelled into a plate containing fresh PBS/0.1% BSA and re-harvested with a different needle. The single cells were expelled into cold lysis buffer (10 mM Tris-HCl [pH 8.3], 50 mM KCl, 1.5 mM MgCl_2_, 5 mM DTT, 0.5% NP-40) and reverse transcribed using Superscript II (Invitrogen) combined with an oligo dT primer. This first strand cDNA product was tailed with A's using terminal deoxynucleotidyl transferase (TdT) and PCR amplified for 35 cycles using the same oligo dT primer. Subsequent gene specific PCR reactions for *pax6*, *chx10* and *cyclin D1* were performed using the primer pairs detailed previously [36].

### Affymetrix array hybridization

Ten micrograms of each single cell cDNA was digested with DNase I (Roche) for 13 minutes at 37°C, heated to 99°C for 15 minutes and biotin labeled using Biotin N6 ddATP (Enzo Biosciences) and TdT (Roche) at 37°C for 1.5 hours. The Affymetrix microarrays were prepared and hybridized using standard Affymetrix protocols [9], [36]. To facilitate comparisons among microarrays, global scaling was performed using the Affymetrix Microarray software (MAS 5.0) and the target intensity was set to 500. The resulting signal data for each probe set was exported as a tab delimited text file and subsequent analyses were performed using Microsoft Excel. The raw and processed Affymetrix data files have been deposited in the NCBI Gene Expression Omnibus (GEO, http://www.ncbi.nlm.nih.gov/geo/) and are accessible through GEO series accession numbers GSE9811 and GSE9812.

### Detection of associated genes

#### Hierarchical clustering

Prior to any clustering analysis, the single cell data were filtered such that any probe set that did not reach a signal level of 1000 in at least one single cell was removed. Hierarchical cluster associations were determined using gene Cluster software [37] and visualized in Treeview [37]. Genes were chosen as clustering closely together by this method if the correlation coefficient for their association was >.75.

#### Fisher's exact test

Probe sets were filtered such that only those that achieved at least a single value of 1000 or greater in at least one single cell were retained. The signal values from all 128 microarrays (includes RPCs from this study, developing ganglion, amacrine and photoreceptor cells [36], bipolar cells [Kim et al, in press], amacrine cells [Cherry et al, in preparation] and Muller glia [Roesch et al, in press]) were binned into 5 equally sized bins (for details on bin number choice see [36]). All probe set pairs were then analyzed for association using the following procedure: First, a contingency table with n rows and n columns was obtained that recorded the joint distribution over bins for a given probe set pair. A P value for significant association was then calculated from this table using Fisher's exact test.

#### Visual inspection in Microsoft Excel

The single cell data were filtered such that signal values less than 1000 were removed. A value of 1000 was chosen as the background level because in general across the data set the Affymetrix algorithm labeled these signals with an absent call. In a limited number of replicate experiments (where the same cDNA was labeled independently more than once), Affymetrix present calls were extremely reproducible in terms of signal value, while the actual signal values for the absent calls varied somewhat widely. Importantly, genes whose signals were labeled as absent in one run were never labeled as present by the Affymetrix algorithm in a replicate run, regardless of their values. To isolate genes with heterogeneous expression patterns in RPCs, the 42 RPCs identified by the classification method described in this study were compared both among themselves and to the developing RGC, AC and PR cells [36].

#### Section in situ hybridization

ISH on retinal cryosections was performed as previously described [38], [39] with the modifications detailed in [36]. A complete list of all the specific ISH probes used in this study and a summary of their expression patterns in presented in Table S8.

#### Dissociated cell in situ hybridization (DISH) and autoradiography

Retinas were dissected from other ocular tissues and incubated as intact explants with [^3^H]-thymidine (5 µCi/µl in DMEM) for 1 hr. For pulse-chase experiments, pregnant mice were injected with [^3^H]-thymidine (10 µCi/g) and harvested after the indicated times. These labeled retinas were washed 3 times in PBS (pH 7.4), dissociated with papain, and plated on poly-D lysine coated slides (10 µg/ml in PBS [Sigma]). Cells were fixed to the slides with 4% paraformaldehyde (PFA) for 10 min. at room temperature, washed twice in PBS (pH 7.4) and dehydrated into 100% methanol. DISH was performed on these cells as previously described [36]. Digoxigenin labeled probes were detected using a combination of anti-digoxigenin-POD (1∶1000, Roche) and a Cy3 tyramide solution (1∶50, PerkinElmer Life Sciences). To quench the first peroxidase reaction, 0.3% hydrogen peroxide (in PBS) was used. Fluorescein-labeled probes were then detected using an anti-fluorescein-POD antibody (1∶1000, Roche) and an Alexa 488-tyramide (1∶100, Molecular Probes). The final reaction was stopped by incubation in 4% PFA for 30 min. The slides were washed in PBS (pH 7.4), DAPI stained, and then allowed to dry. To visualize the [^3^H]-thymidine, slides were dipped in an autoradiography emulsion (NTB2, Kodak) and exposed in the dark for either 2 days (in vitro labeling) or 2 weeks (in vivo labeling). The slides were subsequently immersed in developer for 2 min. (D19, Kodak), rinsed in dH_2_O, and incubated in fixer (Kodak) for 20 min. Finally, the slides were washed in dH_2_O for 20 min. and mounted.

## Results and Discussion

### Single Cell Isolation

Retinas were collected from six different stages of mouse development, ranging from E12.5, which is just after the onset of retinal neurogenesis, to P0, near the end of neurogenesis. These times were chosen to maximize the number of retinal progenitor cells (RPCs) harvested and profiled, as well as to provide newborn neurons and more differentiated cells for comparison. Previous experiments in mouse and rat led to the prediction that between 90% (E12.5) and 30% (P0) of the cells present at these times should be RPCs [18], [40]. In addition, almost all of the ganglion cells, horizontal cells, and cone photoreceptors are born during these timepoints and many rod photoreceptors and amacrine cells are generated as well (reviewed in [19])[41]. Although many bipolar cells and Müller glia are generated after P0, there are still a significant number of these cell types that are born at P0 [41], [42]. These birthdating experiments indicate that cells isolated between E12.5 and P0 can hypothetically capture the gene expression programs in RPCs that lead to the generation of all the retinal cell types, as well as the gene expression profiles of many of the maturing retinal cell types.

The retinas were dissected and dissociated to individual cells using papain and single cells were harvested using a capillary pipette drawn into a fine needle (see Materials and Methods and [36] for more details). Since retinal cells at these early stages of development do not show definitive morphology, cells were chosen at random and the post hoc strategy described below was used to retrospectively classify the cells as RPCs, transitional cells, or postmitotic neurons. The isolated single cells were lysed and subjected to a 35 cycle RT-PCR based protocol that was previously shown to generate a sufficient amount of cDNA (10–20 µg) for hybridization on Affymetrix microarrays [9], [36], [43]. Additionally, medium samples were removed from the dish containing the dissociated cells and subjected to the same RT-PCR method to control for the presence of cDNAs from lysed cells within the media.

The quality of the resulting single cell cDNA products was assessed using several methods. First, the cDNA was examined on an agarose gel and those preparations that contained products ranging from 500 bp to 2 kb were subjected to further testing (data not shown). Media controls consistently failed to exhibit significant cDNA smears (data not shown). To further evaluate the quality of the single cell cDNA mixtures, gene specific RT-PCR was performed using three genes known to be highly expressed in the developing retina (*pax6*, *chx10* and *cyclin D1*) [27], [44], [45], [46]. Robust bands were detected in those preparations that displayed the most robust cDNA smears and bands were routinely not observed with the media controls (data not shown). One final, more comprehensive, approach was utilized to assess the single cell cDNAs. Ten micrograms of cDNA were labeled with Cy5 and hybridized to cDNA microarrays spotted in our laboratory [28], [47]. These microarrays contained ∼12,000 ESTs derived from the retina (Bento Soares, University of Iowa) and many retinal expressed genes from our laboratory [47]. Many of the transcripts spotted on these cDNA microarrays showed significant signal when hybridized with cDNA from the single cells, whereas amplifications from media controls did not show signals above background (data not shown). Taken together, these data demonstrated that more than 50% of isolated single retinal cells yielded cDNA of sufficient quantity and quality for more complete gene expression profiling on Affymetrix microarrays.

Ten micrograms of cDNA from each single cell to be profiled was DNase treated, labeled with biotinylated ddATP using TdT, and hybridized to Affymetrix mouse 430 2.0 oligonucleotide arrays using standard Affymetrix protocols (see Materials and Methods and [9], [36] for full details). These arrays allowed over 34,000 transcripts, nearly the entire mouse transcriptome, to be potentially sampled for expression in each single cell. The data were collected and normalized using Affymetrix Microarray software (MAS 5.0). For each probe set on the array, signal levels, present/absent calls, and detection p-values were exported to Microsoft Excel (Table S1). In addition, the Affymetrix data files for each single cell profiled in this study have been deposited in the NCBI Gene Expression Omnibus (GEO, http://www.ncbi.nlm.nih.gov/geo/) and are accessible through GEO series accession numbers GSE9811 and GSE9812.

In order to gain an appreciation for the level of noise, or unexpected signals, in the single cell preparations, the signals for genes predicted not to be expressed within retinal cells were examined. Signals for immunoglobulin genes (n = 7), cytokeratins (n = 36), and muscle genes (n = 9) were examined. Signal levels were almost invariably <1000 (Table S2 and Figure S1) and were correspondingly denoted as absent by the Affymetrix algorithm. For the five genes that showed significant signal, signal was not present in very many cells (Table S2 and Figure S1). Since previous work on these genes did not assess retinal expression, it was not clear whether these signals were due to transcriptional activity of these loci in the retina, or were false positives due to the single cell method. Examination of SAGE tags for these genes showed that expression was detected for 2 of these 5 genes, suggesting that at least in these cases, there was bona fide retinal expression. To further assess the robustness of the single cell data, the levels of housekeeping genes were examined. It was not clear which genes should be used for this test, as several studies have demonstrated that housekeeping gene expression is highly variable, as assayed by microarray [48], SAGE [27], [49], or other profiling methods [50], [51], [52]. Similar variability has been observed using preparations of retinal tissue [27], [28]. Nonetheless, a list of control genes for RNA preparations generated by two commercial vendors (Qiagen and Superarray Bioscience) was used for this test (Table S3 and Figure S2). Many of the genes were observed to have somewhat consistent levels of expression across the RPCs (Table S3 and Figure S2), consistent with their role as housekeeping genes. However, while each of the genes was present in at least one single cell, for several of them there was a great deal of variability in their expression (Table S3 and Figure S2). It was not the case that a particular cell expressed low levels of all of these genes, as might be predicted for a poor cDNA preparation. Even within a single cell, a high degree of variability in the level of signal for individual genes was observed (Figure S2). These data are consistent with the observations made using total tissue preparations, and thus are not a result of the single cell method. It is more likely the case that assumptions about consistent expression levels of many housekeeping genes are incorrect.

### Retinal progenitor cell classification

The main goal of this study was the determination of the degree of heterogeneity in gene expression among individual RPCs. The first step in this process was to identify a particular transcriptional profile as arising from a cycling RPC instead of a postmitotic retinal neuron. To accomplish this, a post hoc classification scheme was devised based upon clusters of co-expressed genes that were centered around previously identified markers of RPCs, RGCs, ACs or rod photoreceptors. Cone photoreceptors and horizontal cells were excluded as none of the single cell profiles examined appeared to have originated from either of these cell types, following inspection of the array profiles for markers of these cell types. This result was not surprising as both cell types are quite rare [41]. The validity of this classification scheme was first tested on the set of cells containing developing RGCs, ACs, and PRs since these cells had already been extensively characterized [36].

To classify a particular single cell as a developing RGC based upon its gene expression profile, genes strongly associated with the RGC marker *neurofilament light* (*NF68*) were determined. The single cell expression profiles used to generate this list of genes included the potential RPCs profiled for this study, developing RGCs and ACs [36], single mature bipolar cells (Kim et al., in press), single mature amacrine cells (Cherry et al., in preparation) and single mature Müller glia (Roesch et al., in press). Using a Fisher's exact test (see Materials and Methods and [36]), the probability that there was a correlation between the distribution of any given gene and *NF68* was calculated. Setting a p-value cutoff of 10^−3^ yielded 81 genes highly associated with *NF68* (Table S4). Many genes previously shown to have significant RGC expression, such as *GAP43* [36], were included in this list. At least one gene known to be expressed in RGCs, *Brn3b*, was not found to be strongly associated with *NF68*. The reason for the absence of an association between these two genes most likely is that temporally *Brn3b* is turned on in newborn RGCs prior to *NF68*. In fact, two of the RPCs (E14 cell B1 and E16 cell F1) identified as transitional cells in the process of deciding upon a final fate (see below) possessed significant levels of *Brn3b*, but were devoid of *NF68*. Since all of the cells profiled in this study were isolated during retinal development, genes with such temporally distinct windows of expression would not necessarily be expected to associate together. However, *NF68* and *Brn3b* were strongly associated by hierarchical clustering [36]. This lack of correlation again demonstrates the increased utility of the Fisher's exact test over other clustering methods for analyzing gene expression data from single cells.

**Figure 1:**
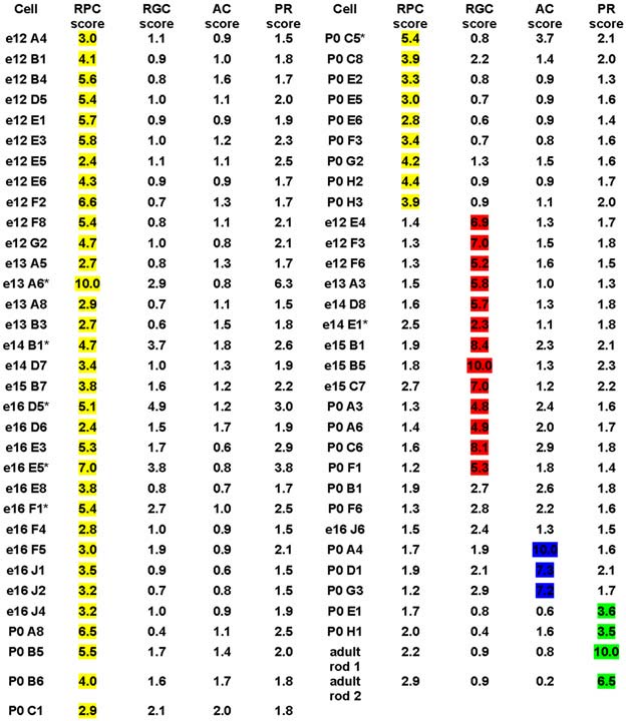
Classification of Retinal Progenitor Cells. A classification scheme was developed to identify RPCs, RGCs, ACs and rod photoreceptors based on the expression of gene clusters. The scaled scores shown are derived from clusters of genes that were associated with known markers for the different cell types by a Fisher's exact test (p<.001). The markers used were *NF68* for RGCs, *TCFAP-2β* for ACs, *Nrl* for PRs and a combination of *Cyclin D1*, *Fgf15*, *Sfrp2* and *Crym* for RPCs.

The relative expression levels for each of the 81 genes associated strongly with *NF68* (as well as *NF68* itself) were calculated by dividing the signal from each single cell by the maximum signal for that gene across the entire data set of single cells. The scaled values for each of the 82 genes within each cell were summed. All sums were then scaled, so that the maximum score was 10, to generate an RGC score for each single cell (Figure 1). This operation was carried out for markers of all cell types on all cells, as described further below. Scaling of the summed scores was required due to the fact that, for each cell type (below), the number of genes defining that cell type differed. An examination of the scores for the 13 previously characterized developing RGCs revealed that, for 12 of these cells the RGC score was considerably higher than the RPC, AC or PR score (Figure 1). For one cell, E14 cell E1, the RPC score and the RGC score were almost the same. Since this cell was a developing RGC and not isolated from adult tissue, the most likely explanation for this result is that this is a cell that was transitioning from an RPC to an RGC. This cell added to the six RPCs designated as transitioning means a total of 7 cells were identified as transitional cells, those having characteristic gene expression of multiple cell types, and these will be discussed in more detail below.

In a similar manner to that used for RGCs, classification scores were generated for ACs and PRs using gene clusters built around the transcription factors *TCFAP-2β* and *Nrl* respectively (see Table S5 and S6 for full clusters). Many of the genes identified as strongly associated with either *TCFAP-2β* or *Nrl* were predicted based upon previous work that characterized them as having either AC expression (*Lrrn3*, *TCFAP-2α* and *Bruno-like 4* for example [36]) or rod photoreceptor cell expression (*Crx*, *IRBP*, *Pde6a*, *Rom1*, and *Tulp1* for example [53]). In the *TCFAP-2β* associated genes, at least one previously known AC gene, *glycine transporter 1*, was not identified (Table S5) because the AC cells isolated in this study were all GABAergic ACs (as assessed by *Gad1* expression [36]). Using these sets of associated genes to generate classification scores revealed that 4 out of 4 rod photoreceptor cells (2 adult and 2 P0) had significantly higher PR scores than the RGCs and ACs (Figure 1). However, the *TCFAP-2β* associated genes only yielded considerably higher AC scores for 3 out of the 6 ACs (Figure 1). This result demonstrates the sensitive nature of this classification scheme since it had been previously noted that these single ACs appeared to fall into 2 distinct classes based upon analysis of their gene expression using other methods [36]. Additionally, one of these groups of 3 ACs scored approximately the same for ACs as they did for RGCs (Figure 1). Again, this points to the robust nature of this classification scheme as these cells were also previously observed to have many similarities in gene expression to developing RGCs [36].

Given the success of this classification scheme in sorting out the different types of retinal neurons, it was used to distinguish the profiles of cycling RPCs from those of the developing, but more committed, retinal cell types. *Cyclin D1* has been characterized as a gene expressed broadly in cycling RPCs [27], [44] and, therefore, this gene was chosen to generate a list of associated genes for classifying profiled single cells as RPCs. The distribution of *cyclin D1* expression was compared pairwise to the signal levels for every other gene on the array across 128 single cell profiles in exactly the same manner as for the RGC, AC and PR markers. This yielded 94 associated genes whose expression was significantly similar in distribution to *cyclin D1* (Table S7). Included in this list were several ribosomal protein genes and other known RPC expressed genes such as *Fgf15* [27]. The relative expression levels for each of these genes and scaled scores were calculated (data not shown). Upon inspection, however, the distribution of these scores was observed to be very narrow owing to the high levels of expression for many of these genes in the profiled single cells and the persistence of many of these transcripts in newborn neurons (data not shown). Therefore, to improve the classification of cycling RPCs, additional gene clusters were added to generate a composite RPC score.

To generate a composite RPC classification score, three additional genes (*Fgf15*, *Sfrp2* and *μ-crystallin*) were chosen to generate gene clusters. These genes have been observed previously in the outer neuroblastic layer (ONBL) of the retina, where the RPCs reside [27]. These 3 genes were also chosen as they together accommodate some of the temporal heterogeneity of the RPCs, as described below. Using the Fisher's exact test and a cutoff p-value of 10^−3^ as before, associated genes were identified for each of these three genes (Table S7). The relative expression levels were calculated and scaled RPC scores generated. As shown in Figure 1, 42 cells displayed a significant RPC score. For 36 of these cells, this score was considerably higher than that for RGC, AC, or PR, establishing these single cell profiles as coming from cycling RPCs (Figure 1). For the additional 6 cells (denoted with an * in Figure 1 and E14 cell E1, see above), while the RPC was the highest, at least one other classification score was significant as well. These cells are most likely transitional cells, RPCs that are in the process of generating a postmitotic daughter and a full analysis of their gene expression will be presented elsewhere (Trimarchi and Cepko, in preparation). Since transcripts expressed in RPCs would not be expected to disappear immediately, it was predicted that some cells would possess profiles containing genes expressed in one or more neuronal cell types, together with RPC genes that are in the process of being downregulated. Such transitional cells are of interest as they provide a window into cells that might still be in the process of deciding upon a final fate [54]. If this state was plastic, it might be revealed through the expression of markers of multiple neuronal cell types.

To assess the utility of this classification scheme relative to a more classical method, the 42 RPC single cell profiles were clustered with the 23 developing or mature RGCs, ACs and PRs using hierarchical clustering. The genes used for hierarchical clustering of these cells were those shown by the Fisher's exact test to be most closely associated with *Cyclin D1*, *Fgf15*, *Sfrp2*, *Crym*, *NF68*, *TCFAP-2β*, and *Nrl* (Tables S4, S5, S6, S7). Hierarchical clustering of these cells showed a definitive separation between the developing neurons and the RPCs (Figure S3). Additionally, the PRs were distinct from the RGCs and ACs (Figure S3). However, the hierarchical clustering method could not distinguish the RGCs from the ACs, whereas the devised classification scheme did, at least for certain AC types, as noted above. A further benefit of this classification scheme over the hierarchical clustering was its ability to identify transitional cells, as the hierarchical clustering did not distinguish these particular RPCs from any others. Additionally, the hierarchical clustering separated the RPCs into several subgroups, one of which contained mainly RPCs isolated at P0. This separation was based upon the cluster of genes that associated strongly with *μ-crystallin* (Table S7) and were expressed only in RPCs during later timepoints (see below). However, two RPCs isolated from earlier timepoints (E12 cell A4 and E13 cell A8) were placed into this cluster by the clustering program despite the fact that these cells did not express this cluster of genes. This observation, coupled with the inability of the hierarchical clustering algorithm to discern RGC profiles from AC profiles makes it difficult to interpret the significance of any further subdivisions of the RPCs.

### Classical and new markers of RPCs

**Figure 2:**
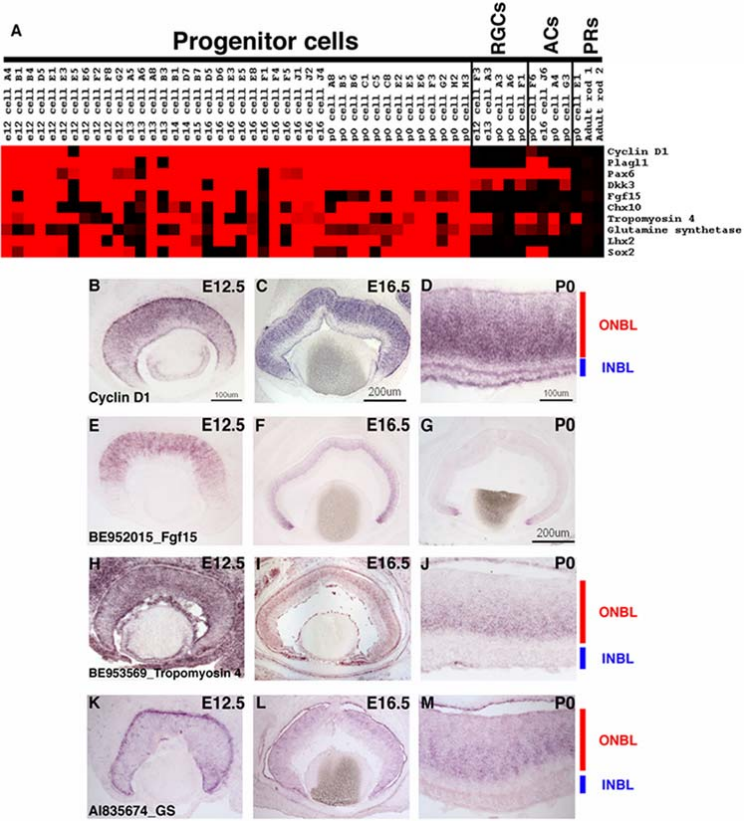
Single cell transcriptional profiles of selected broadly expressed RPC genes. (A) A heatmap was generated using Treeview software and displays the expression of the designated genes in 42 RPCs, 5 RGCs, 4 ACs, and 3 PRs. The intensities from the Affymetrix signals have been scaled such that signals of 10,000 or greater are colored bright red, signals below 1,000 (called absent by Affymetrix software) are black, and signals in between 10,000 and 1,000 are graded according to their signal value. (B) ISH was performed on retinal cryosections at E12.5 (B, E, H, K), E16.5 (C, F, I, L), and P0 (D, G, J, M) using the following probes: *Cyclin D1* [27] (B–D), BE952015 [*Fgf15*] (E–G), BE953569 [*Tropomyosin 4*] (H–J) and AI835674 [*Glutamine synthetase* (*GS*)] (K–M). Representative scale bars are shown in the first panel of each column. All subsequent panels in that column are at the same scale unless otherwise indicated. Cellular laminae of the developing retina are diagrammed with the colored bars.

Immunohistochemistry and/or ISH are standard techniques used to determine the distribution of particular cell types. However, these methods cannot resolve the simultaneous expression of a large number of genes, which would allow one to determine how often it was that a particular cell expressed all or most of the known markers used to define that cell type. The single cell profiles for RPCs were thus examined to determine the distribution of classical markers of RPCs in the single cell profiles. All except one (41/42, see e12 cell E5) of the single cells classified as an RPC displayed high levels of *cyclin D1* expression, while most of the developing RGCs, ACs and PRs did not possess *cyclin D1* transcripts (Figure 2A). Mice deficient for any one of the transcription factors *Pax6*, *Chx10,* or *Sox2* have been shown to exhibit severe eye defects, traceable to these factors playing crucial roles in RPC proliferation and maintenance [55], [56], [57]. Given their prominent role in retinal development and RPC biology, the expression of these three genes was examined in the single RPC profiles. In accordance with their important developmental roles, all three were widely expressed in the single RPC profiles (Figure 2A). One potentially interesting feature of their expression was that while they were observed in >75% of RPCs, they were not seen in all of the RPCs (Figure 2A). This observation could either be a false negative result stemming from some aspect of the single cell technique, or alternatively, it could point to a previously unappreciated degree of gene expression heterogeneity in RPCs.

**Figure 3:**
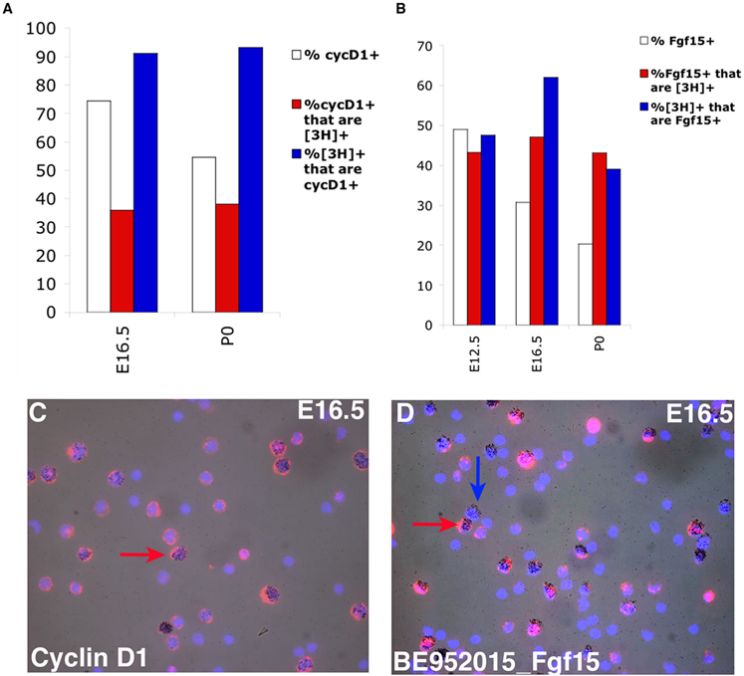
Examination of broadly expressed RPC genes by dissociated cell *in situ* hybridization (DISH). DISH was performed using either *Cyclin D1* or *Fgf15* probes. E12.5, E16.5 or P0 retinas were explanted, labeled with [^3^H]-thymidine for 1 hour, dissociated and probed for either (A, C) *Cyclin D1* [27] or (B, D) BE952015 [*Fgf15*]. The graphs in A and B show the percentages of total DAPI^+^ cells that were also positive for either *Cyclin D1* (A) or *Fgf15* (B) as well as the percentages of cells positive for either gene in relation to the population of [^3^H]-thymidine^+^ cells. Additionally, the percentage of [^3^H]-thymidine^+^ cells that express each gene is shown. The red arrows in C and D indicate cells that are positive for both [^3^H]-thymidine and for the probed gene. The blue arrow in D indicates a cell that is [^3^H]-thymidine^+^, but negative for *Fgf15*.

In order to develop assays to more fully explore the potential heterogeneity of gene expression in RPCs, two ISH methods were used to examine expression, with an initial test using a probe for *Cyclin D1*, the gene expressed in the highest percentage of RPCs profiled. Confirmation of expression in RPCs was possible on section ISH since RPCs reside in a distinctive layer, the outer neuroblastic layer (ONBL), though they are not the only cell type in the ONBL as migrating neurons also are present in this layer. In addition, expression of a gene in an RPC is detectable in cells acutely labeled with [^3^H]-thymidine, using the quantitative method of dissociated ISH (DISH). ISH using a *cyclin D1* riboprobe on retinal cryosections from E12.5, E16.5 and P0 revealed strong staining in the ONBL, and diminished staining in the inner neuroblastic layer (INBL), where most postmitotic neurons are located (Figure 2B–D). DISH was performed on dissociated retinas that had been incubated with [^3^H]-thymidine for 1 hour before dissociation. This method primarily labels cells in S-phase and to a minor degree, cells in the early portion of G2. DISH performed at E16.5 or P0 with a *cyclin D1* riboprobe showed that greater than 90% of [^3^H]-thymidine^+^ cells were also *cyclin D1* ^+^ (Figure 3A,C), indicating that most S-phase progenitor cells express *cyclin D1*. As a control, *GAP43*, a gene expressed in developing RGCs [36], was never observed in [^3^H]-thymidine^+^ cells (data not shown). Additionally, only ∼1/3 of *cyclin D1* ^+^ cells were [^3^H]-thymidine^+^ (Figure 3A,C), consistent with the microarray data that suggested that *cyclin D1* was present in RPCs in other cell cycle phases, as well as S phase. DISH for other genes broadly expressed in RPCs showed that 93% of [^3^H]-thymidine^+^ cells at E16.5 expressed *Pax6* and 93% of [^3^H]-thymidine^+^ cells expressed *Chx10*. It should be noted that the microarray data reflects expression patterns of RPCs in all cell cycle phases, not just S phase, so an exact match of the percentage of RPCs positive for a given gene in the microarray analysis would not necessarily be expected in the [^3^H]-thymidine/DISH experiments. Nonetheless, these data demonstrate that a very high percentage of at least S phase RPCs express these genes. At the same time, they also show that a clear minority of [^3^H]-thymidine^+^ cells was negative for these RPC markers. This finding corroborates the microarray results, where not all RPCs were positive for these RPC marker genes (see Figure 2A). Although the possibility exists that the [^3^H]-thymidine^+^ population does not appear to be 100% for these genes for technical reasons, DISH performed for *Ubiquitin B* at E16.5 did show that 100% of the [^3^H]-thymidine^+^ cells expressed *Ubiquitin B*, demonstrating that at least for one probe this degree of co-staining was achievable. Taken together, these data indicate that most, but not all, RPCs do indeed express classical markers of RPCs at all times. The mild degree of heterogeneity observed likely reflects some dynamic processes within these cells. A similar suggestion was made based upon observations of the expression of a transgene encoded by a Chx10 BAC, in which the onset of expression was not uniform throughout the retina [58].

In addition to examining the RPC profiles for the expression of classical RPC marker genes, we wished to identify new genes that were consistently expressed among most or all RPCs, and thus could serve as new markers of RPCs and perhaps reveal new findings regarding RPC biology. To identify such genes, three different approaches were used. First, the RPC genes identified using the Fisher's exact test that were used for classifying the single cells were examined. Second, hierarchical clustering was performed on the set of single cells including the 42 RPCs and the 21 developing neurons previously characterized [36] using Gene Cluster software [37]. Finally, genes with potentially interesting RPC expression patterns were identified by visual inspection of the microarray data in Microsoft Excel (for details see Materials and Methods and [36]). Many different types of genes were found to be broadly expressed in the single RPCs and some representative examples are shown in the heatmap generated by Treeview in Figure 2A. The types of genes identified ranged from transcription factors (*Plagl1* and *Lhx2*) to secreted molecules (*Fgf15* and *Dkk3*). The presence of these transcripts in many of the RPCs continued to demonstrate the robustness of the single cell profiling method since these genes have been shown to be expressed in the retina by other means [27], [46], [59], [60]. As observed earlier for genes such as *Sox2* and *Chx10*, these additional RPC genes were broadly expressed throughout individual RPCs (i.e. found in >50% of RPCs and in many cases >75% of RPCs), but most of these genes also were not observed in all RPCs, displaying some heterogeneity of expression.

To investigate the heterogeneous expression of the newly identified broadly expressed RPC markers, section ISH and DISH were performed for one such gene, *Fgf15*. According to the microarray data, *Fgf15* was expressed in 9 out of 11 *cyclin D1* ^+^ E12.5 RPCs, 9 out of 11 E16.5 cells and 10 out of 13 P0 cells (Figure 2A). Section ISH was performed at E12.5, E16.5 and P0. *Fgf15* was expressed in the ONBL at E12.5 (Figure 2E) and E16.5 (Figure 2F), but was already less intense in the center of the retina at E16.5 (Figure 2F), despite the continued presence of RPCs at this stage. By P0, strong staining for *Fgf15* was only observed in the most peripheral portion of the ONBL (Figure 2G). At all of the timepoints observed, it appeared that *Fgf15* expression was much patchier than that of *cyclin D1* (compare Figure 2B to Figure 2E for instance), confirming the heterogeneity in *Fgf15* expression. DISH also was performed on E12.5, E16.5 or P0 retinas that had first been labeled for 1 hr with [^3^H]-thymidine. When compared with the results for *cyclin D1*, the percentage of *Fgf15* ^+^ cells was consistently lower, whether among the entire population of retinal cells or among the [^3^H]-thymidine^+^ population (compare Figure 3A and 3B). In fact, the expression of *Fgf15* was heterogeneously expressed in the S phase population of RPCs at all the timepoints examined (Figure 3D). Two color DISH on P0 retinal cells revealed that while >90% of *Fgf15* ^+^ cells also expressed *cyclin D1*, only 50% of *cyclin D1* ^+^ cells expressed *Fgf15*. The section ISH and DISH provide a confirmation that the heterogeneity of *Fgf15* expression shown in the microarray analysis is unlikely to be a consequence of the single cell method, but reveals bona fide heterogeneity in expression among RPCs, even from a given age and in the same portion of the cell cycle, and even for a gene that is generally broadly expressed in RPCs.

In addition to the validation of previously characterized RPC markers, the single cell method revealed the expression of new genes broadly expressed in RPCs that were not previously characterized in the retina, such as *tropomyosin 4* (*Tpm4*). Interestingly, it also revealed expression in RPCs for genes previously recognized solely for their expression in mature cell types, but not RPCs, such as *glutamine synthetase* (*GS*). ISH on retinal cryosections from E12.5, E16.5 and P0 mice with riboprobes for *Tpm4* (Figure 2H–J) or *GS* (Figure 2K–M) showed strong staining in a significant portion on the ONBL, confirming that this gene is expressed in many RPCs. Tropomyosins are actin binding proteins that have been shown to play roles in the establishment of neuronal polarity [61], but a role for these proteins in the developing retina has not been explored. GS expression has been well characterized in Müller glia cells of the adult retina [62], [63], but again a role for this enzyme in the developing retina has not been elucidated.

### Temporal changes in RPC gene expression

**Figure 4:**
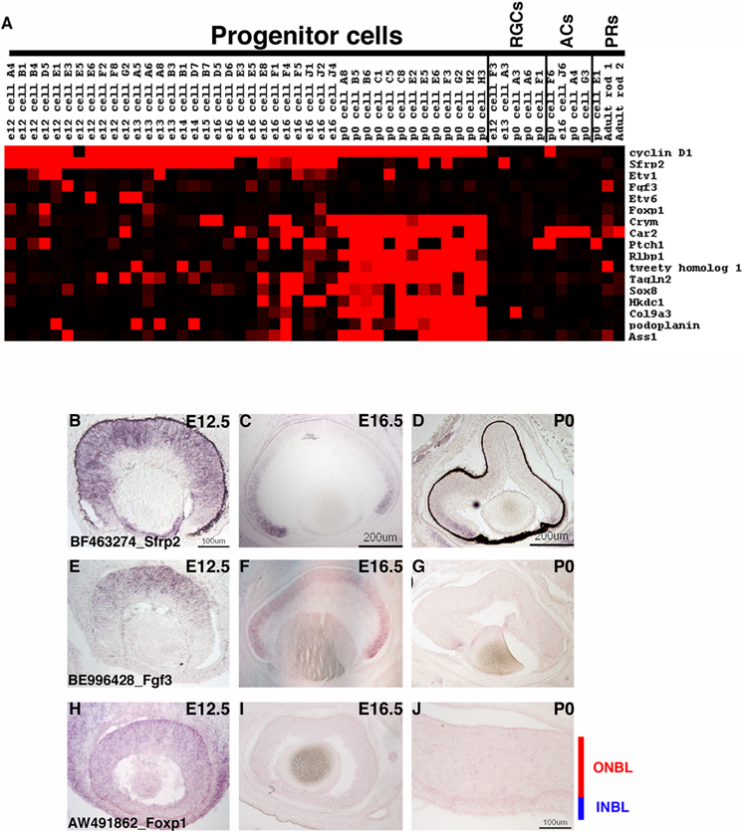
Expression of temporally regulated transcripts in RPCs. (A) A Treeview generated heatmap showing the expression of transcripts that are enriched in RPCs at early developmental stages (top portion) and those that are enriched at later developmental stages (bottom portion). *Cyclin D1* expression is depicted for comparison. ISH for genes expressed in RPCs at early developmental stages was performed at E12.5 (B, E, H), E16.5 (C, F, I) and P0 (D, G, J). The probes used were (B–D) BF463274 [*Sfrp2*], (E–G) BE996428 [*Fgf3*], and (H–J) AW491862 [*Foxp1*]. Representative scale bars are shown in the first panel of each column. All subsequent panels in that column are at the same scale unless otherwise indicated. Cellular laminae of the developing retina are diagrammed with the colored bars.

Classical birthdating experiments have shown that the different retinal cell fates are produced at different times during the course of retinal development [16], [17], [18]. In addition, heterochronic mixing experiments demonstrated that RPCs could only produce the temporally appropriate cell fates when placed in an environment of a different developmental stage [20], [21], [22]. Given these results, it was expected that a comparison of the single cell profiles from E12.5 RPCs to those of P0 RPCs would reveal genes that were expressed primarily in either early or late RPCs. *Secreted frizzled-related protein 2* (*Sfrp2*), a gene previously identified in a retina SAGE screen [27], was in fact only observed in early RPCs and its expression was almost completely extinguished by P0 (Figure 4A–D). Examining gene clusters generated around *Sfrp2* either by hierarchical clustering methods (data not shown) or by using a Fisher's exact test (Table S7) did reveal some genes with correlated expressions in RPCs, but consistently failed to yield genes with a close match for the temporal expression pattern of *Sfrp2*. Most of the associated genes were expressed in RPCs at timepoints beyond when *Sfrp2* was detected (Table S7 and data not shown). Comparing the gene expression profiles of E12.5 RPCs and P0 RPCs by visual inspection in Microsoft Excel, however, did reveal several candidate genes whose expression appeared mainly confined to early RPCs (Figure 4A). There were not a large number of these genes and their expression was restricted to a small subset of the profiled RPCs, unlike *Sfrp2,* which was more broadly expressed in early RPCs. Interestingly, three of these early genes were transcription factors (*Foxp1*, *Etv1* and *Etv6*) and one was a cell signaling molecule (*Fgf3*). To confirm the temporal gene expression pattern of these transcripts, ISH was performed on E12.5, E16.5 and P0 retinal cryosections. These experiments demonstrated that while these genes appeared to be present in fewer cells than *Sfrp2*, the kinetics of their expression did parallel that of *Sfrp2* (Figure 4E–J and data not shown). *Fgf3* was the most similar to *Sfrp2* by ISH with significant staining in the ONBL at E12.5 (Figure 4E) and staining only in the most peripheral portion of the ONBL by P0 (Figure 4G). However, *Fgf3* staining was more localized toward the center of the retina, while *Sfrp2* staining extended more peripherally (compare Figure 4B and 4E). Given that retinal neurogenesis initiates in the center and spreads to the periphery, this difference in spatial patterns between *Sfrp2* and *Fgf3* may point to roles in the initiation/progression of neurogenesis. In the chick and zebrafish, Fgf3, in conjunction with Fgf8, plays an important role in controlling the onset of neurogenesis [64]. In the single cell RPC profiles from mouse, *Fgf8* was not detected (Table S1). However, it is still tempting to speculate that Fgfs might be playing a similar role in the mouse since both *Fgf15* and *Fgf9* are expressed in RPCs along with *Fgf3*. In addition, *Fgf12* and *Fgf13* have been found in the postmitotic neurons of the INBL [36]. The reasons why different cells in the developing retina express distinct combinations of growth factor genes remains to be explained, but these expression patterns point to a surprising amount of gene expression heterogeneity in the RPC population, even for genes with a presumed similarity in function.

**Figure 5:**
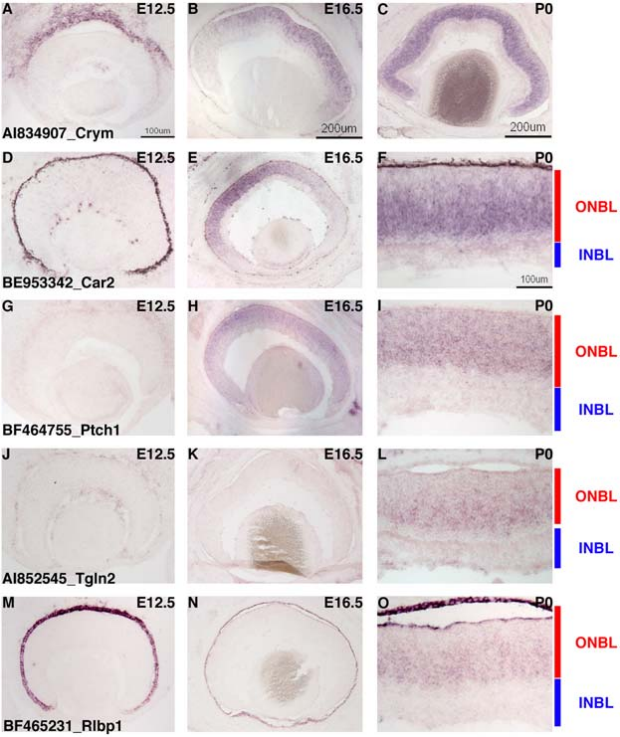
Expression of temporally regulated transcripts in RPCs. ISH on retinal cryosections was performed at three stages: E12.5 (A, D, G, J, M), E16.5 (B, E, H, K, N) and P0 (C, F, I, L, O). The following probes were used: (A–C) AI834907 *[μ-crystallin*], (D–F) BE953342 [*Carbonic anhydrase 2*], (G–I) BF464755 [*Patched1*], (J–L) AI852545 [*Transgelin 2*], and (M–O) BF465231 [*Retinaldehyde binding protein 1*]. Representative scale bars are shown in the first panel of each column. All subsequent panels in that column are at the same scale unless otherwise indicated. Cellular laminae of the developing retina are diagrammed with the colored bars.

In contrast to small number of genes expressed only at early timepoints in the developing retina, a tightly regulated cluster of late expressed genes emerged, centered around *μ-crystallin*. This cluster was apparent in both hierarchical clustering methods and a Fisher's exact test (Table S7) and a representation of the *μ-crystallin* cluster is shown in the bottom portion of the heatmap in Figure 4A. To examine the expression of these genes more thoroughly, section ISH was performed at E12.5, E16.5 and P0. Consistent with the microarray results, many of these genes were not detected in E12.5 retinal cryosections (Figure 5). At least one, *retinaldehyde binding protein 1* (*Rlbp1*), was detected strongly in the pigment epithelium layer (RPE) at E12.5 (Figure 5M), but not in the retina itself. At E16.5, the kinetics of expression of these genes split into two groups. The first set was turned on by E16.5 and this group was represented by *Crym* (Figure 5B), *Carbonic anhydrase 2* (*Car2*) (Figure 5E) and *Patched 1* (*Ptch1*) (Figure 5H). Interestingly, *Car2* was clearly detected in the ONBL in these ISH experiments as well as in the microarrays, whereas in previous studies *Car2* expression in RPCs was contentious [27], [62]. The second group of late expressed RPC genes were not detected by ISH until P0 (Figure 5J–L and M–O).

The precise functions of these early and late expressed RPC genes are unclear at present. Since the early expressed genes either impinge upon signaling pathways (*Sfrp2* and *Fgf3*) or are transcription factors (*Etv1*, *Etv6* and *Foxp1*), one can envision these genes playing important roles in early retinal development. Both the Ets factor family in general and *Foxp1* specifically have been shown to play critical roles during the development of the hematopoietic system, so these factors have important functions in uncommitted cells in other systems [65], [66]. Since the expression of these genes is quite heterogeneous, it will be of interest to use the regulatory sequences from the promoters of these genes as reporters to probe the fates of cells expressing each of these early expressed genes. For the late expressed genes, it is intriguing that these genes are more broadly expressed and form a tighter cluster than the early genes. This might be indicative of a shift in competence state. However, if that is true, it is curious that only one of these late expressed genes is a transcription factor (*Sox8*). Sox8 has been implicated in a role for oligodendrocyte development [67], [68] and since Müller glia are one of the last retinal cell types generated, it may play a similar role here. The other genes either have no known function as yet or have been shown to play either enzymatic or structural roles in other organisms [69], [70], [71]. Given the regulation of the gene expression kinetics of these temporally expressed retinal transcripts, though, it seems likely that they may play important roles in the RPCs that give rise to the later born retinal cell types.

### Single RPCs display extensive heterogeneity in gene expression

**Figure 6:**
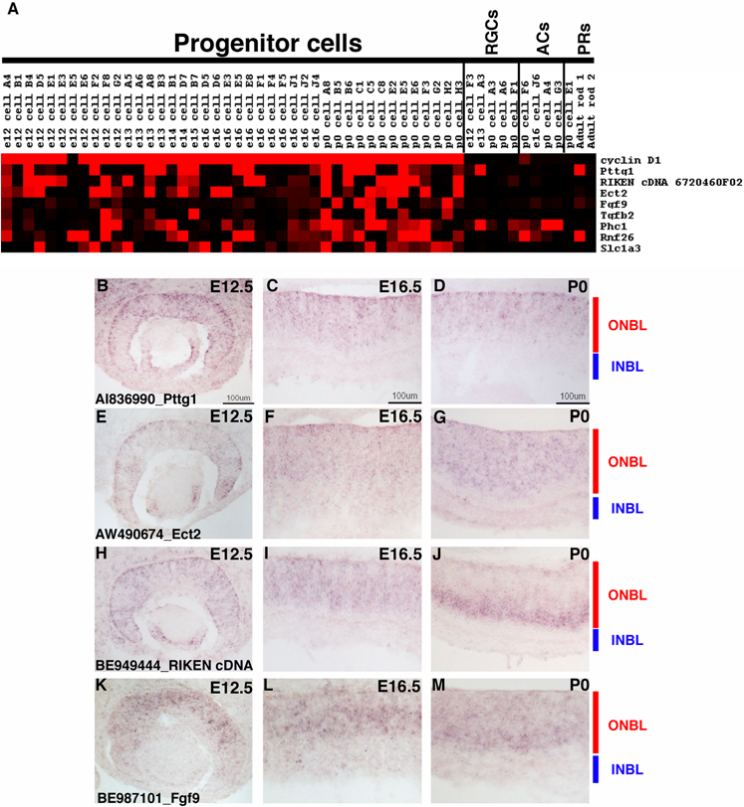
Expression of genes in subsets of RPCs. (A) A Treeview generated heatmap displaying the expression of genes found in subsets of the RPC profiles. *Cyclin D1* expression is depicted for comparison. ISH was performed on retinal cryosections at E12.5 (B, E, H, K), E16.5 (C, F, I, L), and P0 (D, G, J, M) using the following probes: (B–D) AI836990 [*Pttg1*], (E–G) AW490674 [*Ect2*], (H–J) BE949444 [*RIKEN cDNA 6720460F02*], and (K–M) BE987101 [*Fgf9*]. Representative scale bars are shown in the first panel of each column. All subsequent panels in that column are at the same scale unless otherwise indicated. Cellular laminae of the developing retina are diagrammed with the colored bars.

**Figure 7:**
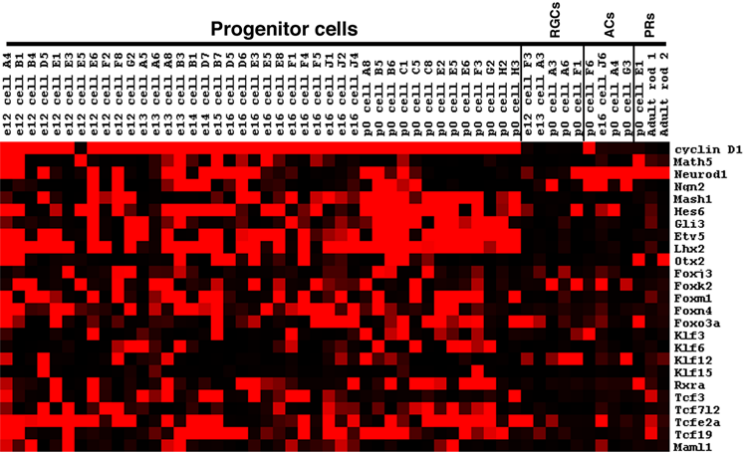
Expression of different transcription factors in RPCs. A Treeview generated heatmap showing the expression of selected transcription factors in single RPCs. *Cyclin D1* expression is depicted for comparison.

The single cell gene expression profiles were examined in Microsoft Excel for genes that were present in relatively small subsets of the 42 identified RPCs. A high number of genes displayed expression in ∼50% or fewer of the RPC profiles (Figure 4A, 6A, 7 and Figure S4). To confirm that the expression of these genes was in fact confined to a subset of RPCs, ISH was performed on retinal cryosections from E12.5, E16.5 and P0 (see Table S8 for a summary of all probes). A riboprobe for *pituitary tumor-transforming gene 1* (*Pttg1*) resulted in staining of only a subset of cells in the ONBL at all the stages examined (Figure 6B–D). This gene has been implicated in the control of cellular proliferation and transformation through a mechanism that is not yet entirely clear, but is believed to involve the control of the oncogene c-myc [72]. While the role of *Pttg1* in retinal development is still unknown, it probably does not involve *c-myc* specifically, since *c-myc* is only found in subsets of developing RGCs in the retina [36]. Another gene that has been linked to tumor progression, *epithelial cell transforming sequence 2* (*Ect2*) [73] was also detected in a subset of RPCs (Figure 6A). Some of these RPCs also expressed *Pttg1*, but some did not, again illustrating the extent of the gene expression heterogeneity in these cells (Figure 6A). ISH confirmed the expression of *Ect2* in subsets of cells in the ONBL across the three stages of mouse retinal development (Figure 6E–G). The presence of many transcripts linked to cellular proliferation and transformation in RPCs most likely reflects the dynamic nature of the control of cell division in a developing tissue. The notion to emerge from these single cell data is the fact that not all of the same control molecules are expressed in the same RPCs. While this might reflect cell cycle differences at the time of harvesting (see below for more discussion), the section ISH patterns for at least *Pttg1* and *Ect2* do not point to expression of these factors in any specific cell cycle phase and instead favor the idea that these RPCs, even from the same timepoint, are distinct in their gene expression profiles.

As previously noted for the broadly expressed RPC genes, the genes observed in smaller subsets of RPCs also represented many different classes. Signaling molecules (*Fgf9* and *Tgfb2*), uncharacterized cDNAs (*RIKEN cDNA 672046F02*, *Rnf26*), polycomb group members (*Phc1*), and a glutamate transporter (*Slc1a3*) are just a few of the different types of genes found in subsets of RPCs (Figure 6A). ISH again confirmed the heterogeneity of expression of these genes, as they were each observed in subsets of cells in the ONBL at the E12.5, E16.5 and P0 (Figure 6H–M and data not shown). A recent study demonstrated that glutamate could contribute to the regulation of cell proliferation in RPCs. Treatment of retinas with glutamate resulted in a decrease in cellular proliferation [74]. Expression of a glutamate transporter in only a subset of RPCs might reflect the ability of that particular subset of RPCs to regulate their proliferation in response to an extrinsic cue, perhaps providing only these specific cells with a link between their environment and their intrinsic transcriptional programs.

### Heterogeneity of transcription factor expression in single RPCs

Transcription factors (TFs) represent an obvious category of genes for influencing numerous processes in RPCs, from driving changes from one competence state to another, to beginning cascades that end in a RPC generating a particular cell type. On the Affymetrix mouse 430 2.0 microarray, there were ∼2400 target sequences annotated as a TF (www.netaffx.com) representing approximately 2000 different TFs. Examination of the array signals for the 42 RPCs revealed that ∼50% of these TFs achieved a present call (>1000) in at least one of the RPCs (Figure S4). Furthermore, ∼700 of these TFs showed a medium to high level of signal (>5000) in at least one of the 42 RPCs (data not shown). These TFs ranged in their frequency of expression, from those showing expression in nearly all 42 of the RPCs (see top 1/3 of Figure S4) to those displaying extensive heterogeneity of expression (3 or 4 out of 42 RPCs) across the RPCs (lower 2/3 of Figure S4 and Figure 7). To attempt to discover coregulated sets of TFs, different clustering methods were used, including hierarchical clustering and a Fisher's exact test. However, none of the methods employed was capable of identifying overlapping TFs that behaved in a coordinated manner. There are most likely several reasons for this result. First, some of the TFs are expressed in very few of the profiled RPCs, making it impossible to correlate their expression with any other genes with any statistical reliability. Second, the available algorithms might not be able to account for the combinatorial nature of the action of TFs. For instance, it is possible that when two TFs are expressed together in the same single cell they lead to a certain cellular outcome. However, these same two factors might also be expressed separately in other single cells. A combination of more sophisticated algorithms and functional studies will be necessary to fully understand the extensive heterogeneity of TF expression in developing RPCs.

**Figure 8:**
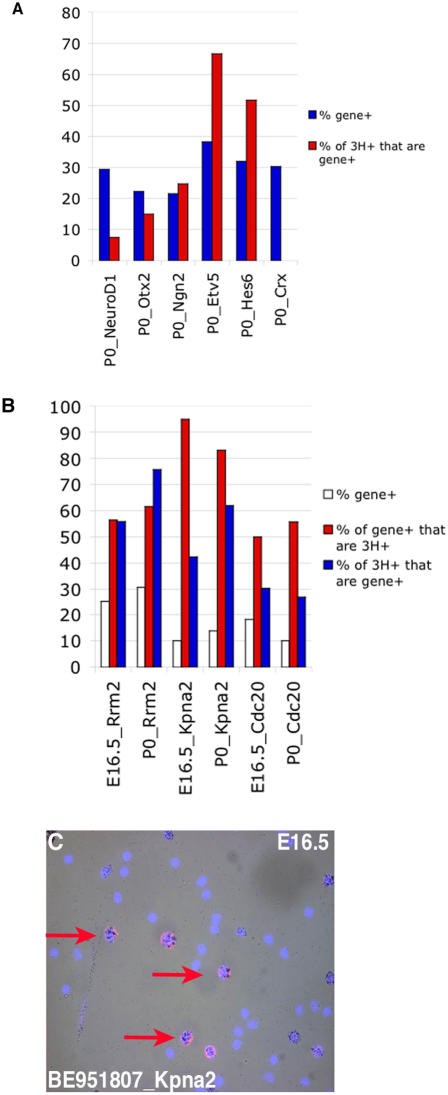
Examination of TFs and cell cycle markers by DISH. E16.5 or P0 retinas were explanted, then labeled with [^3^H]-thymidine for 1 hour, and dissociated. (A) The slides were probed for *NeuroD1* [132], *Otx2* [27], BQ178789 [*Ngn2*], BE996421 [*Etv5*], AW048812 [*Hes6*] or *Crx* [133]. The graphs show the percentages of total DAPI^+^ cells that were also positive for the individual TFs (red bars) and the percentages of [^3^H]-thymidine^+^ cells that express each TF (blue bars). (B) The slides were probed for BE984641 [*Rrm2*], BE981507 [*Kpna2*] or BE988025 [*Cdc20*]. The graphs show the percentages of total DAPI^+^ cells that were also positive for the cell cycle genes (white bars), the percentages of cells positive for the cell cycle genes in relation to the population of [^3^H]-thymidine^+^ cells (red bars), and the percentages of [^3^H]-thymidine^+^ cells that express each TF (blue bars). (C) A representative field from a dissociated E16.5 retina that was labeled with [^3^H]-thymidine and probed with BE981507 [*Kpna2*]. The red arrows indicate three *Kpna2* ^+^ cells that are also [^3^H]-thymidine^+^.

Neurogenic basic helix-loop-helix (bHLH) transcription factors have been shown to play crucial roles in the generation of many postmitotic retinal cell types [75], [76], [77]. Recently the loss of one bHLH, Math5, was shown to lead to deficiencies in cell cycle progression in RPCs, revealing a possible additional coordinating role for this class of TF in RPCs [78]. Understanding the mechanism of action of these bHLH factors requires a detailed knowledge of their expression patterns. In the 42 single RPC profiles, the neurogenic bHLH genes were found in subsets of cells (Figure 7). To verify that these bHLH factors were expressed in RPCs, retinas were pulse labeled with [^3^H]-thymidine and DISH was performed for *Math5* or *NeuroD1*. At E16.5, 18% of [^3^H]-thymidine^+^ cells were Math5^+^ while at P0 6% of [^3^H]-thymidine^+^ cells were NeuroD1^+^ and 25% were *Ngn2* ^+^ (Figure 8A). These results indicate that while the bulk of cycling RPCs are not expressing these bHLHs, these genes most likely begin their expression either in late S phase or early G2. Interestingly, when one bHLH transcript was observed in an RPC, other bHLH transcripts were present as well, and some RPCs expressed as many as 4 different bHLH genes (Figure 7). For example, at least three cells at three different timepoints expressed significant levels of *Math5*, *Ngn2* and *NeuroD1* (see E13 cell B3, E16 cell D6 and P0 cell C1). This result was surprising given previous reports that both *Ngn2* and *NeuroD1* were suppressed in Math5 expressing cells [78]. However, these previous conclusions were based upon upregulation of these bHLHs in the absence of Math5 [78] and not a direct observation of their co-expression. Interestingly, single cell RT-PCR in the chick retina revealed that a few cells could co-express certain bHLHs [79]. Additionally, 7 RPC single cell profiles showed co-expression of *Ngn2* and *Mash1*, including cells isolated from 3 different timepoints (Figure 7). Again this result is in contrast to previous observations that *Ngn2* and *Mash1* were never expressed in overlapping cells [55]. However, as before, this prior result was not based upon direct detection of *Ngn2* and *Mash1* transcripts, but instead relied upon GFP-based reporters for both genes [55]. It is possible that these reporters did not fully recapitulate the entire spectrum of expression for these genes, perhaps due to differences in the regulation of transcription or translation, as has been shown for certain homeobox TFs in Xenopus [80]. These single cell profiles demonstrate the expression of multiple neurogenic bHLHs in single RPCs and suggest that the interplay among these TFs is perhaps not as simple as previously postulated. These data provide a potential explanation for the observed redundancy of these bHLH factors in retinal development [81]. Furthermore, Xenopus NeuroD1 has been shown to be regulated by phosphorylation and if similar regulatory mechanisms exist in the mouse [82], this could provide a method for independently controlling bHLHs that are co-expressed.

**Figure 9:**
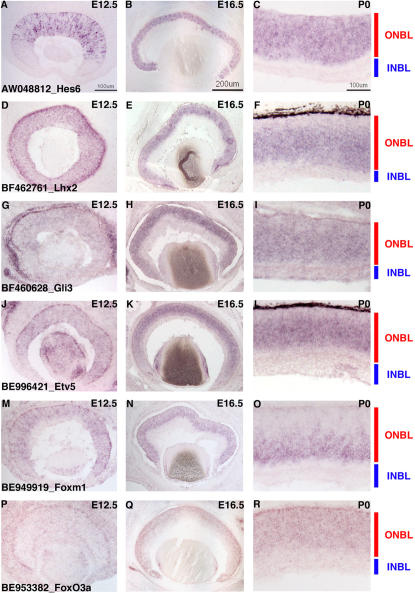
TF expression in RPCs. ISH on retinal cryosections was performed at three stages: E12.5 (A, D, G, J, M, P), E16.5 (B, E, H, K, N, Q) and P0 (C, F, I, L, O, R). The following probes were used: (A–C) AW048812 [*Hes6*], (D–F) BF462761 [*Lhx2*], (G–I) BF460628 [*Gli3*], (J–L) BE996421 [*Etv5*], (M–O) BE949919 [*Foxm1*], and (P–R) BE953382 [*FoxO3A*]. Representative scale bars are shown in the first panel of each column. All subsequent panels in that column are at the same scale unless otherwise indicated. Cellular laminae of the developing retina are diagrammed with the colored bars.

*Hairy and enhancer of split 6* (*Hes6*) is an additional bHLH gene whose protein product antagonizes the activity of other Hes family members and thereby facilitates the action of neurogenic bHLHs [83]. The single cell expression profiles showed *Hes6* expression in RPCs across developmental time and revealed a significant overlap in expression with *Mash1* at P0 (Figure 7). *In situ* hybridizations with a *Hes6* riboprobe revealed a subset of cells in the ONBL stained at E12.5 and E16.5 (Figure 9A,B), with broader ONBL staining at P0 (Figure 9C). DISH performed on [^3^H]-thymidine pulse labeled retinas showed that 25% of [^3^H]-thymidine^+^ cells also stained for *Hes6*, confirming the presence of this transcript in RPCs (Figure 8A). As this is a time when many RPCs are producing postmitotic neurons, it is consistent with previous work showing that Hes6 has a positive role in this process [83].

Many homeodomain (HD) containing transcription factors have been found to play crucial roles in retinal development [32], [56], [84], [85]. In the analysis of TF expression among single RPCs, many HD containing TFs were observed. Some of these TFs showed a broad expression pattern in the single RPCs (for example *Pax6*, *Chx10*, *Sox, Lhx2, Six3*) (See Figure 2, 7, 9D–F and Figure S4), while others showed more heterogeneity of expression (*Otx2*, *Rax*, *Six5*). *Otx2* expression was confirmed to begin in RPCs by DISH on [^3^H]-thymidine pulse labeled retinas. In these experiments, 15% of [^3^H]-thymidine^+^ cells were also *Otx2* ^+^ (Figure 8A), indicating that the expression of *Otx2* mRNA most likely begins in the late S to early G2 phase of the cell cycle. Pulse-chase experiments at E18.5 further validated this idea by showing that the number of [^3^H]-thymidine^+^ cells that were also *Otx2* ^+^ increased from 9% 4 hours after labeling to 28% by 24 hours after labeling. Interestingly, *Crx*, another HD containing TF that is involved in photoreceptor development and maintenance [86], [87], showed similar, but slightly delayed kinetics of onset to that of Otx2. *Crx* was not present in [^3^H]-thymidine^+^ cells after a 1 hour pulse (Figure 8A) or a 4 hour chase, but was only first observed in 15% of [^3^H]-thymidine^+^ cells 24 hours after labeling. This observation fits with the predicted regulation of Crx by Otx2 [88].

Two additional TFs, *Gli3* and *Etv5*, exhibited a pattern in the single RPC profiles that was somewhat similar to *Mash1* and *Lhx2* in that there appeared to be more RPCs at P0 expressing these genes that at the earlier timepoints (Figure 7). However, this correlation was not exact and it was difficult to ascertain the precise number of cells expressing these genes at different timepoints on section *in situ* hybridizations (see Figure 9G–L). In DISH experiments, only the riboprobe for *Etv5* yielded significant signal and showed that *Etv5* was in ∼2/3 of the [^3^H]-thymidine^+^ population of RPCs at P0 (Figure 8A). Additionally, these TFs are part of much larger families of factors and many other family members were found in subsets of RPCs as well (Figure 4A and Figure S4). Therefore, at present any possible overlapping roles for these factors in currently unknown. However, recently, *Gli3* was shown to have a genetic interaction with *Pax6* in the developing retina [89] demonstrating that the interplay among all these TFs is probably critically important for retinal development.

The forkhead/winged helix family of transcription factors is considerable in number and these genes have been shown to play important roles in development through the control of cellular proliferation, apoptosis and metabolism [90], [91], [92]. There were numerous forkhead transcription factors expressed in the single cell profiles of RPCs and many of these TFs showed profound heterogeneity in their expression (Figure 4A, 7 and Figure S4). These forkhead factors ranged from those for which there is little known about their functional roles in general and nothing understood of their part in retinal development (*Foxj3* and *Foxk2*) to *Foxn4*, which has been shown to play a crucial role in the ability of RPCs to generate amacrine and horizontal cells [93]. *Foxm1* was also found in a subset of RPCs and ISH revealed expression of this in the ONBL (Figure 9M–O). Intriguingly, at P0, *Foxm1* was confined to the vitreal side of the ONBL where S phase RPCs are located (Figure 9O). In serum starved cells induced to re-enter the cell cycle, *Foxm1* mRNA has been shown to initiate at the onset of S phase and remain on from that point [94]. It is interesting that *Foxm1* may be more tightly regulated in the developing retina, but its expression is consistent with a role in cell cycle control. *Foxo3a* was another family member expressed in subsets of RPCs (Figure 7). Although the *Foxo3a* transcript was detected in cells from early and later developmental timepoints, there were more single RPCs expressing this gene at P0 (Figure 7). Section ISH confirmed this later expression, as signal was observed in more cells in the ONBL at P0 (Figure 9R) than at E12.5 (Figure 9P). The significance of this *Foxo3a* expression in the retina is unclear. In other organisms and contexts, *Foxo3a* has been shown to induce either cell cycle arrest or apoptosis [95], [96]. While it is conceivable that Foxo3a is sensitizing the later retina to apoptotic signals, Foxo transcription factors in general have been linked to the cyclin kinase inhibitor p27Kip1 [92]. Therefore, given that more RPCs at P0 are generating postmitotic daughter cells and p27Kip1 is involved in cell cycle exit in the retina [31], perhaps Foxo3a plays an upstream role in the decision to generate a postmitotic daughter cell.

Many additional TF families are present in the single cell profiles, with different family members represented in distinct subsets of RPCs. For instance, there were at least 5 different Krüppel-like factors (KLFs) detected in subsets of RPCs (Figure 7 and Figure S4). KLF15 has previously been shown to be capable of inhibiting the rhodopsin promoter [97], [98], but otherwise, no function has been ascribed to these factors during murine retinal development. The fact that these KLFs are expressed in RPCs in the mouse is interesting given the important role played by Krüppel in the temporal progression of neuroblasts in *Drosophila* [24]. It has also been shown that Castor, the final gene in the Drosophila cascade, is expressed in photoreceptors in mouse retina [27]. Further examination of the single cell gene expression profiles revealed the presence of both an *ikaros-like zinc finger* (*Ikzf5*) in subsets of RPCs and several POU domain containing genes (Figure S4). The presence of such homologs for other genes in the Drosophila neuroblast cascade in subsets of RPCs during mouse retinal development suggests that a cascade similar to that in *Drosophila* might be playing a role in the progression of competence states in the murine retina as well.

**Figure 10:**
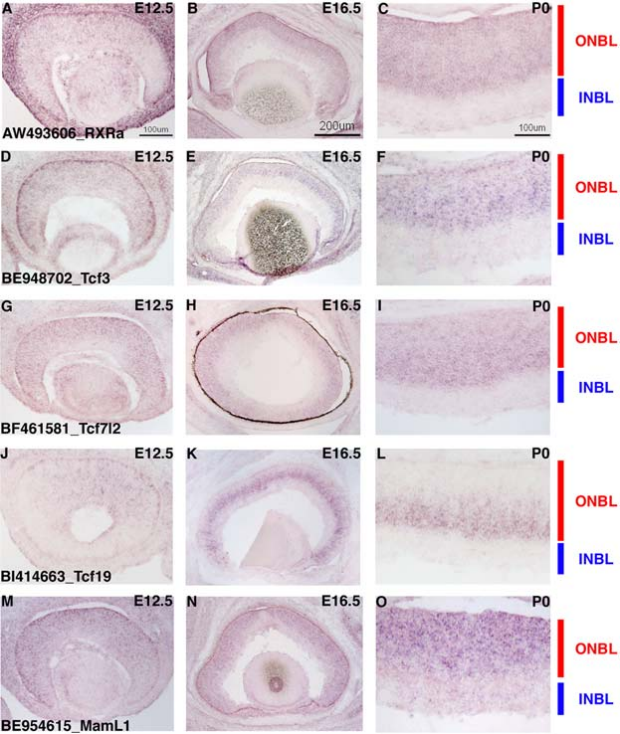
TF expression in RPCs. ISH on retinal cryosections was performed at three stages: E12.5 (A, D, G, J, M), E16.5 (B, E, H, K, N) and P0 (C, F, I, L, O). The following probes were used: (A–C) AW493606 *[RXRα*], (D–F) BE948702 [*Tcf3*], (G–I) BF461581 [*Tcf7l2*], (J–L) BI414663 [*Tcf19*], and (M–O) BE954615 [*MamL1*]. Representative scale bars are shown in the first panel of each column. All subsequent panels in that column are at the same scale unless otherwise indicated. Cellular laminae of the developing retina are diagrammed with the colored bars.

One final TF family expressed in subsets of RPC profiles is the nuclear hormone receptor family of gene regulators. Many members of this large family were observed in distinct subsets of RPCs (Figure S4), including both orphan nuclear receptors such as the CoupTFs (*Nr2f1* and *Nr2f2*) and those for which the hormone is characterized such as *RXRα* (Figure 7, 10A–C). This latter gene is interesting in light of the critical role that retinoic acid signaling plays in dorsoventral patterning of the chick and zebrafish retinas [99], [100], as well as the expression of rod genes, including rhodopsin [101], [102], [103]. Another set of TFs expressed in RPC subsets is the Tcfs that are downstream of the Wnt signaling pathway (Figure 7, 10D–I). *Tcf3* was present in more RPC profiles from early timepoints, while *Tcf7l2* was detected more in P0 RPCs (Figure 7). The possible significance of this early/late expression difference is unclear. Also, the function of these TFs in retinal development remains elusive since the Wnt factors themselves were not strongly detected in the retina (data not shown and [27]) and Wnt inhibitors such as *Sfrp2* were found, at least in the early retina (Figure 4). Finally, some uncharacterized TFs were also identified as expressed in RPCs. One of these factors, *Tcf19* was found in a small subset of RPCs at E12.5 (Figure 7, 10J) and then an increasing subset at subsequent stages (Figure 7, 10K–L). At P0, *Tcf19* transcripts were localized near the vitreal edge of the ONBL (Figure 10J), suggesting this gene may be cell cycle regulated (see below). In total, many different classes of TFs were found expressed in subsets of RPCs throughout retinal development. The challenge in the future will be to use these single cell data in concert with functional studies to understand the precise combinations of TFs that RPCs use to generate all the distinct subtypes of retinal neurons.

### Cell cycle gene transcripts in single RPCs

**Figure 11:**
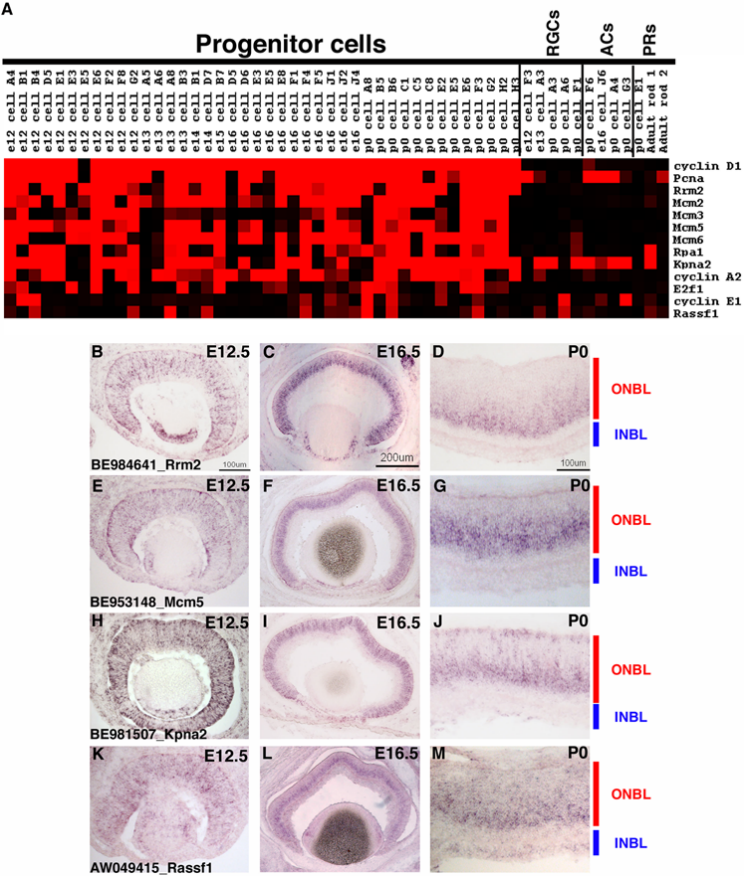
Expression of G1 and S phase cell cycle genes in RPCs. (A) A Treeview generated heatmap displaying the RPC expression of genes previously shown to play roles in either the G1 or S phases of the cell cycle. *Cyclin D1* expression is depicted for comparison. ISH was performed on retinal cryosections at E12.5 (B, E, H, K), E16.5 (C, F, I, L), and P0 (D, G, J, M) using the following probes: (B–D) BE984641 [*Rrm2*], (E–G) BE953148 [*Mcm5*], (H–J) BE981507 [*Kpna2*], and (K–M) AW049415 [*Rassf1*]. Representative scale bars are shown in the first panel of each column. All subsequent panels in that column are at the same scale unless otherwise indicated. Cellular laminae of the developing retina are diagrammed with the colored bars.

One potential explanation for at least some of the observed heterogeneity of gene expression among RPCs is that these cells were not synchronized with respect to the cell cycle and, therefore, most likely exhibit cell cycle associated differences in gene expression. To begin to explore cell cycle differences in gene expression as one possible cause of RPC heterogeneity, genes that had been identified in other settings as correlated with particular phases of the cell cycle were examined for variations in the single RPC profiles. These genes were divided into two groups, G1/S and G2/M, based upon their reported expression, which has been assayed primarily in cell culture. The heatmap shown in Figure 11A depicts a representative sample of the G1/S group of genes assembled from the literature [104], [105], [106], [107], [108], [109]. Genes such as *PCNA*, *Rrm2* and the *Mcm*s (2-6), whose protein products play important roles in DNA replication [110], [111], were observed in a significant subset of RPCs (Figure 11A). *Rrm2*, for example, was observed in 76% (32/42) of the profiled RPCs (Figure 11A). ISH on retinal cryosections confirmed that these genes were expressed in the ONBL (Figure 11B–G and data not shown). In the developing retina, the movement of RPCs is coordinated with the cell cycle so that mitosis occurs at the most scleral edge of the retina, just adjacent to the RPE, and S phase occurs toward the vitreal side of the ONBL [16], [112]. The two gap phases, G1 and G2, occur in the intervening space. Closer inspection of the section ISH patterns for *Rrm2* and *Mcm5* revealed that these genes are more strongly expressed toward the vitreal surface than the scleral surface (Figure 11, especially D and G), indicating they are predominantly detected in S phase cells. At E12.5, the expression pattern of these genes was more scattered throughout the ONBL (Figure 11B, E) likely reflecting the observation that the precise migration patterns of RPCs with respect to the cell cycle do not occur at this early stage (Trimarchi and Cepko, unpublished observations) [78]. DISH conducted for *Rrm2* showed that 56% of *Rrm2* ^+^ cells were [^3^H]-thymidine^+^ at E16.5 and at P0 this number increased to 61% (Figure 8B). These data indicate that *Rrm2* expression is enriched in S phase cells. However, the microarray data predicted a higher number of cells should express *Rrm2* than was observed in the ISH experiments (Figure 11A). These results most likely indicate that *Rrm2* is expressed in both the G1 and S phases of the cell cycle, but at significantly higher levels in S phase, such that the detection by ISH picks up mostly S phase cells. This would be akin to the situation in serum starved and restimulated fibroblasts where *Rrm2* (and many other G1/S genes) was observed at lower, but detectable, levels during G1, with expression increasing significantly at the G1/S transition and into S phase [113]. In the current set of experiments, the single cell profiling is likely more sensitive than the *in situ* methods and therefore is able to detect even low levels of these transcripts in other cell cycle phases.

Through the use of the different clustering methods employed in this study, some genes were uncovered as potentially cell cycle regulated that were not previously well characterized with respect to the cell cycle. In addition, their expression patterns in the developing retina were completely unknown. One such gene was *Karyopherin alpha 2* (*Kpna2*). *Kpna2* was observed in a subset of RPCs (Figure 11A). ISH on retinal cryosections revealed an expression pattern in a scattered subset of ONBL cells at E12.5 (Figure 11H) that progressed to a staining pattern that was concentrated on the vitreal side of the ONBL where the S phase cells reside at P0 (Figure 11J). At E16.5, the expression of *Kpna2* was in a repeated pattern of radial stripes across the retina (Figure 11I). The striped expression pattern is not a predicted pattern, but it may indicate a dynamic regulation of *Kpna2* that is coordinated with neighboring cells. Neighboring cells might share a history of recent cell divisions and thus might be somewhat synchronized with respect to the cell cycle. DISH for *Kpna2* revealed that this gene is almost exclusively expressed in S phase, as 95% of all *Kpna2* ^+^ cells at P0 were also [^3^H]-thymidine^+^ after a 1 hour pulse (Figure 8B,C). The protein product of this gene has been linked to the nuclear import of a protein important for DNA repair and checkpoint function [104], [114]. It is possible that this novel finding of restriction of *Kpna2* expression to cells almost exclusively in S phase points to an important role in regulation of the normal cell cycle in a developing tissue. In addition to this insight into cell cycle function, this observation means that *Kpna2* can be used as a marker for RPCs in S phase in future studies.

One cell cycle gene that was found in the fewest RPCs and might, therefore, be tightly regulated in its cell cycle expression was *Cyclin E1* (Figure 11A). Section ISH for *Cyclin E1* demonstrated that while it showed weak signal in the retina, it was expressed in a small subset of cells near the vitreal surface of the ONBL (data not shown). Hierarchical clustering experiments revealed that the gene *Rassf1* was significantly correlated with *Cyclin E1* (data not shown). ISH on retinal cryosections revealed a similar expression pattern to *Cyclin E1*, both in the intensity of signal and in location (Figure 11K–M and data not shown). While *Rassf1* has been shown to be a tumor suppressor and to affect the ras pathway, its exact function, especially in development, remains poorly understood [115]. Additionally, in tissue culture experiments, Rassf1 has been shown to be capable of impinging on the regulation of the cell cycle at multiple points [115], but an interaction with Cyclin E1 is yet to be explored.

**Figure 12:**
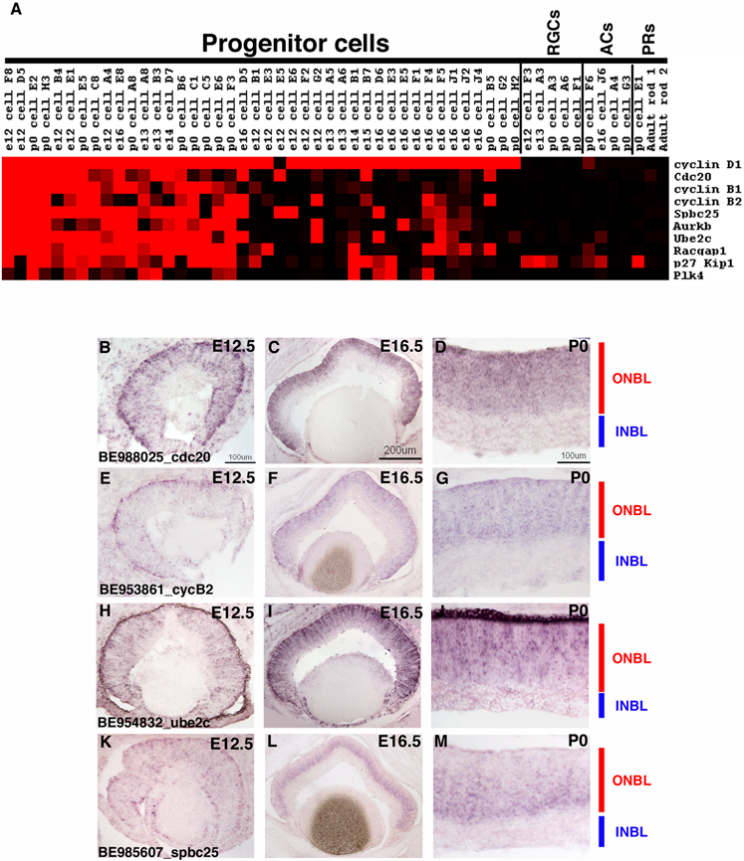
Expression of G2/M phase cell cycle genes in RPCs. (A) A Treeview generated heatmap displaying the RPC expression of genes previously shown to play a role in G2/M portion of the cell cycle. *Cyclin D1* expression is depicted for comparison. ISH was performed on retinal cryosections at E12.5 (B, E, H, K), E16.5 (C, F, I, L), and P0 (D, G, J, M) using the following probes: (B–D) BE988025 [*Cdc20*], (E–G) BE953861 [*Cyclin B2*], (H–J) BE985607 [*Spbc25*], and (K–M) BE954832 [*Ube2c*]. Representative scale bars are shown in the first panel of each column. All subsequent panels in that column are at the same scale unless otherwise indicated. Cellular laminae of the developing retina are diagrammed with the colored bars.

The analysis described above for G1/S phase genes was also done for genes that had previously been shown to have their expression concentrated in the G2 phase of the cell cycle [116], [117], [118], [119], [120]. The heatmap in Figure 12A shows some genes that are representative of the G2/M class. These genes were present in a smaller number of RPCs than those associated with the G1/S phases. As reported by Young [121], RPCs spend less time in G2/M than they do in G1/S, and thus fewer cells will show the G2/M pattern of gene expression than the G1/S pattern. Many RPCs showed robust expression of markers for both G1/S and G2/M (note E12 cell A4, E13 cell B3 and P0 cell F3 as examples in Figure 11A and Figure 12A). These results may point to a tighter transcriptional regulation of genes that play a role in G2/M than those genes involved in the G1 and S phases. Section ISH for some of these G2/M genes revealed some variation in the expression patterns (Figure 12B–M). *Cdc20* was found in subsets of cells in the ONBL at E12.5 (Figure 12B), a radial like pattern at E16.5 (Figure 12C), and throughout the ONBL at P0 (Figure 12D). At all timepoints, though, the expression did appear to be enriched toward the scleral side of the ONBL, where mitosis occurs, but it was never absent from the vitreal side, indicating the possibility of broader expression. DISH performed at both E16.5 and P0 showed significant overlap between [^3^H]-thymidine and *Cdc20* after a 1 hour pulse (Figure 8B), showing that the expression of this gene most likely begins while cells are still in S phase. However, the overall percentage of cells expressing *Cdc20* was always much lower than those expressing a G1/S marker such as *Rrm2* (10% for *Cdc20* versus 30% for *Rrm2* at P0, based upon DISH). *Cyclin B1* and *Cyclin B2* showed a similar expression pattern to that of *Cdc20*, with the exception of being more biased toward the scleral edge of the retina, especially at earlier stages such as E12.5 and E16.5 (Figure 12E–G and data not shown). Ubiquitination and subsequent degradation of many substrates is important for driving cells through anaphase and allowing the completion of mitosis [122]. One enzyme that participates in the ubiquitination is Ube2c and this gene also was found strongly associated with other characterized G2/M markers (Table S9). Section ISH revealed that *Ube2c* was in a subset of cells in the ONBL at all three stages examined (Figure 12H–J) and also displayed a pattern of radial stripes reminiscent to *Cdc20* at E16.5 (Compare Figure 12C with 12I). Surprisingly, *Spbc25*, a component of the kinetochore, used to set up and maintain proper chromosome alignment during mitosis, displayed more intense staining toward the vitreal (S phase) edge of the ONBL than the scleral (M phase) edge (Figure 12K–M). This may indicate that even some of the G2/M class of genes is less tightly regulated in the developing retina than previously appreciated, and/or that there is differential regulation of the RNA and the protein. Finally, the cyclin dependent kinase inhibitor *P27Kip1* was also found strongly associated with G2/M marker genes by hierarchical clustering (data not shown). This suggests that P27Kip1 is involved in the normal transition from G2 through M in RPCs and this is most likely a strong factor as to why in P27^−/−^ mice there is a cell cycle defect, but no corresponding perturbation of the distribution of retinal cell fates [31].

**Figure 13:**
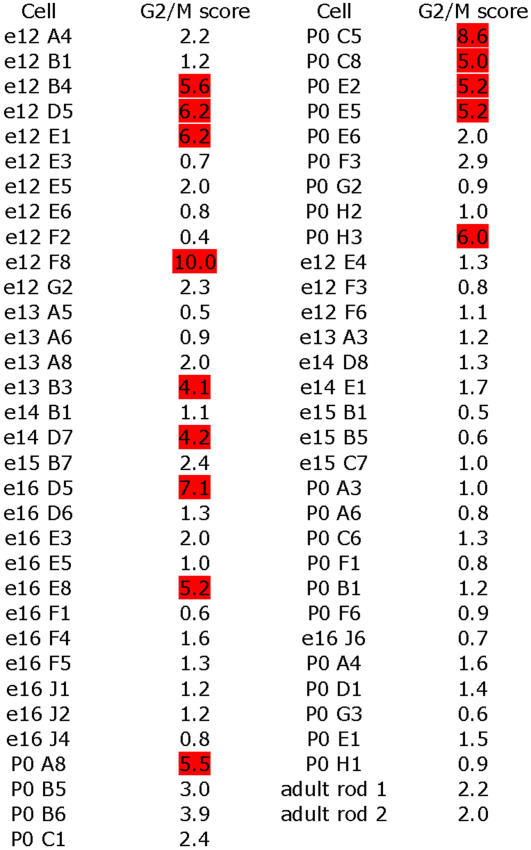
Classification of G2/M progenitors. A classification scheme was developed to identify RPCs in the G2/M phases of the cell cycle. The scaled scores shown are derived from clusters of genes that were associated with the G2/M markers *Cdc20*, *Aurkb* and *Ube2c* by a Fisher's exact test (p<.001).

**Figure 14:**
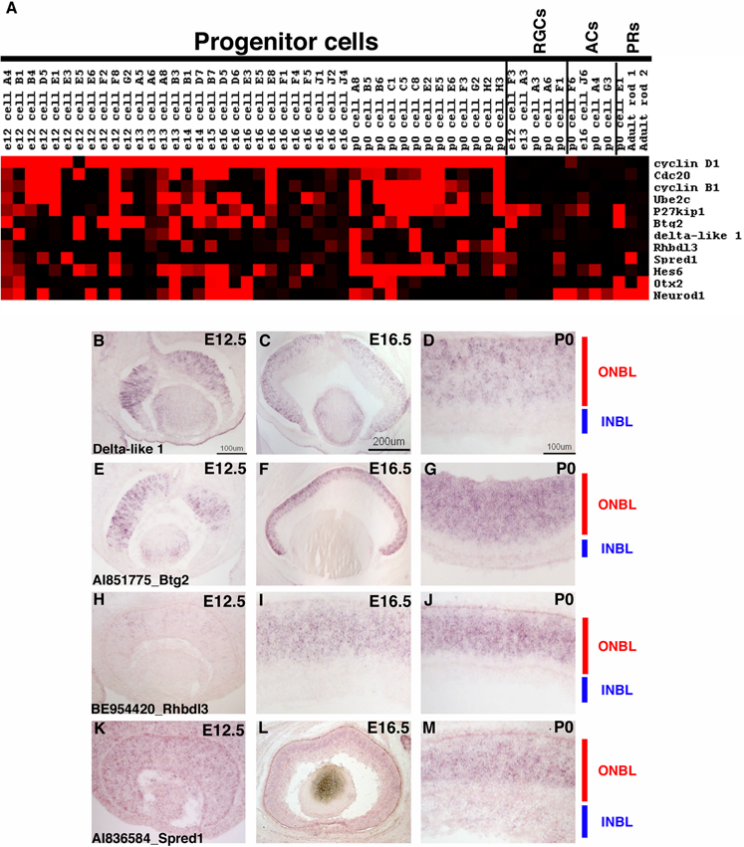
Expression of genes that potentially play a role in cell cycle exit. (A) A Treeview generated heatmap displaying the RPC expression of genes that were isolated due to their expression in subsets of G2/M cells. The expression of *Cyclin D1*, G2/M genes (*Cdc20*, *Cyclin B1*, *Ube2c*, and *P27Kip1*) and some TFs (*Hes6*, *Otx2* and *NeuroD1*) are depicted for comparison. ISH was performed on retinal cryosections at E12.5 (B, E, H, K), E16.5 (C, F, I, L), and P0 (D, G, J, M) using the following probes: (B–D) Delta-like 1 [123] (E–G) AI851775 [*Btg2*], (H–J) BE954420 [*Rhbdl3*], and (K–M) AI836584 [*Spred1*]. Representative scale bars are shown in the first panel of each column. All subsequent panels in that column are at the same scale unless otherwise indicated. Cellular laminae of the developing retina are diagrammed with the colored bars.

Exit from the cell cycle is intimately coordinated with the cell fate decision making process in RPCs [2]. Since the G2/M phases of the cell cycle would most likely be when this link would occur, it was of interest to identify those RPCs with the most characteristic gene expression of G2/M. The classification scheme described above for the identification of cell types was adopted to generate G2/M scores for the RPCs, transitional cells and postmitotic neurons. These scores were a composite based upon the clusters of genes generated around three G2/M markers, *Cdc20*, *Aurora kinase B* (*AurkB*), and *Ube2c* (Table S9). Fourteen of the 42 RPCs displayed high scores for G2/M while none of the postmitotic neurons scored highly (Figure 13). Only 2/6 of the RPCs denoted as transitional cells scored highly for G2/M markers (Figure 13), suggesting that these cells are most likely in different windows of the transition from RPC to postmitotic neuron. Examination of the RPCs with high G2/M gene expression was performed with the hopes of identifying novel genes involved in the process of exiting from the cell cycle. Both hierarchical and Fisher's exact test based clustering methods yielded many significantly associated genes, but given the nature of these genes (many kinesins and microtubule associated proteins), the majority of them probably play generic roles in cytokinesis and other mitotic processes (Table S9 and data not shown). Visual inspection of the single cell profiles in Microsoft Excel, with an extra focus on the cells receiving the highest G2/M scores, revealed several interesting candidate genes that may play a role in the ability of an RPC to exit from the cell cycle (Figure 14A). The notch ligand, *delta-like 1(Dll1)*, whose expression had previously been shown to be well correlated with the timing of retinal neurogenesis [123], was found to be highly expressed in several G2/M cells (see P0 cell A8, P0 cell C8 and P0 cell H3 for examples, Figure 14A). Section ISH showed *Dll1* staining in subsets of cells in the ONBL at all three stages (Figure 14B–D) and DISH performed with P0 retinas that were labeled with [^3^H]-thymidine for 1 hour revealed that 1/3 of *Dll1* ^+^ cells were also [^3^H]-thymidine^+^. These data are consistent with expression of *Dll1* at the right place and the right time to be correlated with the production of a postmitotic cell(s) by an RPC. Three additional genes (*B-cell translocation gene 2* [*Btg2*], *Rhomboid veinlet-like 3* [*Rhbdl3*], and *Sprouty protein with EVH-1 domain 1* [*Spred1*]) were discovered in subsets of G2/M RPCs (Figure 14A). *Btg2* was found in subsets of ONBL cells at E12.5 (Figure 14E) and E16.5 (Figure 14F), but its expression appeared to become more broadly expressed at P0 by section ISH (Figure 12G). This result is consistent with *Btg2* playing a role in cell cycle exit since many more retinal neurons are being generated at P0 than at E12.5. Btg2 has been shown to enhance neural differentiation upon overexpression in PC12 cells [124] and its expression in the neural tube correlates with those cells that will generate a postmitotic neuron [125], further suggesting a role for this gene in the control of cell cycle exit in the retina. Both *Rhbdl3* and *Spred1* were found in subsets of RPCs and both were in more RPC profiles at P0 than at the earlier stages (Figure 14A). Section ISH for both genes confirmed that their expression increased as the number of retinal neurons generated increased (Figure 14H–M). Neither gene has been extensively characterized in general, nor does any information exist as to the possible functions of these genes during retinal development. Drosophila homologues of *Rhbdl3* have been shown to modulate both the EGF pathway and the notch pathway and, in that manner, play specific roles in the cell fate specification of neuroblasts [126], [127]. Alternatively, *Spred1* has been shown to be a negative regulator of the ras pathway and perturbation of *Spred1* function interfered with neural differentiation in tissue culture [128]. Given that both of these genes have been shown to be possible regulators of important pathways that are also at work in the developing retina, they make excellent candidates for regulators of the cell cycle exit and cell fate decision-making processes of RPCs. The fact that these different candidate genes are expressed in distinct RPCs suggests that the machinery utilized by RPCs to exit the cell cycle may vary among exiting cells.

### Conclusions

This study has examined the transcriptomes of 42 individual RPCs across multiple developmental timepoints and has uncovered considerable heterogeneity of gene expression among these cells. The microarray results were validated and extended by using a combination of section ISH and DISH to an extent not previously reached by the few other single cell studies [6], [7], [8], [9], [10]. The degree to which single RPCs varied in their gene expression was not previously appreciated using profiling methods that employed sampling of the entire retina, which contains multiple different cell types, even at a single timepoint. One striking aspect of the observed RPC heterogeneity was the significant contribution that TFs made to the gene expression differences. Many different TF families were represented by a large number of distinct family members in individual RPCs. In addition, cell cycle markers, especially those that play a role in the G2/M phases of the cell cycle, were observed in only a subset of RPCs. Although there was no readily apparent link between the heterogeneous expression of TFs and different cell cycle markers, genes potentially involved in cell cycle exit were found in G2/M RPCs. The heterogeneity of RPC expression of these genes points to the intriguing possibility that different RPCs may use distinct mechanisms for exiting from the cell cycle. Future studies will be required to address whether these distinct mechanisms can be linked to specific cell fates.

As more studies on the gene expression of single cells appear, the one commonality that is shared by all of them is the discovery of a greater degree of gene expression heterogeneity than had been previously believed to exist. This finding has held true for multiple experimental systems ranging from simple populations of engineered *Escherichia coli* [129], to a cell line treated with retinoic acid [130] and even to individual hematopoietic stem cells [10]. The data presented here demonstrate for the first time the enormous heterogeneity of gene expression in progenitor cells as a tissue develops. The functional consequences of these gene expression differences will not be fully understood without more experimentation, but several models are conceivable. It may be that the heterogeneity of gene expression is rendering certain subsets of RPCs competent to respond to particular environmental cues and this would bias these cells toward producing specific cell fates. Alternatively, the differences might not be played out in differential responses to environmental cues, but might reflect intrinsic differences that drive cell fate decisions, and/or cell cycle decisions, relatively independent of the environment. A contrasting model would be that the fluctuations in gene expression, which could be stochastic and/or regulated, might not be meaningful in terms of progenitor behavior. . This latter model would make RPCs similar to HL-60 cells that are initially quite different in their responses to retinoic acid, but eventually reach the same final cellular destination [130]. The generation of specific reporters of gene expression history, and the perturbation of gene function [131] will allow for a greater understanding of how the retina develops.

## Supporting Information

Figure S1 Assessment of unexpected signals in the single cell profiles. A Treeview generated heatmap showing the lack of expression of immunoglobulin genes, keratin genes, and muscle genes in the majority of single RPCs gene expression profiles.
(9.52 MB TIF)

Figure S2 Assessment of housekeeping gene levels in the single cell profiles. A Treeview generated heatmap showing the expression of housekeeping genes in single RPCs.
(3.99 MB TIF)

Figure S3 Classification of single cells. Hierarchical clustering was used to generate a dendrogram of RPCs, RGCs, ACs, and PRs.
(6.05 MB TIF)

Figure S4 Expression of TFs in RPCs. A Treeview generated heatmap showing the expression of transcription factors in single RPCs across many different transcription factor families.
(9.52 MB TIF)

Table S1 Affymetrix array data for all of the single cells profiled in this study. Scaled Affymetrix signal values (see Materials and Methods), present/absent calls, and detection p-values are shown for each probe set for each RPC cell profiled in this study.
(84.74 MB XLS)

Table S2 Assessment of unexpected signals in the single cell profiles. Signal levels are shown for genes that were predicted not to be expressed in single RPCs. In the instances where multiple Affymetrix probe sets were present for the same gene, the maximum signal level was chosen.
(0.04 MB XLS)

Table S3 Assessment of housekeeping gene levels in the single cell profiles. Signal levels are shown for a list of housekeeping genes as determined from available lists (Qiagen and Superarray Bioscience).
(0.03 MB XLS)

Table S4 Genes used for RGC classification. Genes are shown that associate with NF68 with a p value <0.001 by a Fisher's exact test along with the corresponding GO information for each gene. The signal levels for these genes were used to generate a GC score for each cell (see Figure 1).
(0.05 MB XLS)

Table S5 Genes used for AC classification. Genes are shown that cluster with TCFAP-2Beta with a p value <0.001 by a Fisher's exact test along with the corresponding GO information for each gene. The signal levels for these genes were used to generate an AC score for each cell (see Figure 1).
(0.04 MB XLS)

Table S6 Genes used for PR classification. Genes are shown that cluster with Nrl with a p value <0.001 by a Fisher's exact test along with the corresponding GO information for each gene. The signal levels for these genes were used to generate a PR score for each cell (see Figure 1).
(0.11 MB XLS)

Table S7 Genes used for RPC classification. Genes are shown that cluster with Cyclin D1, Fgf15, Sfrp2 or Crym with a p value <0.001 by a Fisher's exact test along with the corresponding GO information for each gene. The signal levels for these genes were used in conjunction to generate a composite RPC score for each cell (see Figure 1).
(0.18 MB XLS)

Table S8 A summary of genes for which in situ hybridizations were performed in this study. For each gene the corresponding cDNA used to generate the ISH riboprobe is listed. A summary of the expression pattern observed at each stage is included. Terms used to describe the in situ patterns: Outer neuroblastic layer/ventricular zone (ONBL), scleral portion of the ONBL (sONBL), vitreal portion of the ONBL (vONBL), retinal pigment epithelium (RPE), not determined (ND).
(0.05 MB XLS)

Table S9 Genes used for G2/M classification. Genes are shown that cluster with Cdc20, AurkB or Ube2c with a p value <0.001 by a Fisher's exact test along with the corresponding GO information for each gene. The signal levels for these genes were used in conjunction to generate a composite G2/M score for each cell (see Figure 13).
(0.04 MB XLS)
